# Bacterial Spectrum of Spontaneously Ruptured Otitis Media in a 7-Year, Longitudinal, Multicenter, Epidemiological Cross-Sectional Study in Germany

**DOI:** 10.3389/fmed.2021.675225

**Published:** 2021-05-20

**Authors:** Matthias Imöhl, Stephanie Perniciaro, Andreas Busse, Mark van der Linden

**Affiliations:** ^1^National Reference Center for Streptococci, Department of Medical Microbiology, Aachen University Hospital, RWTH Aachen University, Aachen, Germany; ^2^Laboratory Diagnostic Center, Aachen University Hospital, RWTH Aachen University, Aachen, Germany; ^3^Pediatric Medical Practice, Tegernsee, Germany

**Keywords:** acute otitis media, bacterial spectrum, vaccination, Germany, etiology

## Abstract

We analyzed middle ear fluid (MEF) and nasopharyngeal swabs (NPS) from spontaneously ruptured acute otitis media (AOM) cases occurring in children under 5 years in Germany. The aim of the study was the assessment of disease burden and bacterial etiology in the era of routine pneumococcal vaccination. Furthermore, we aimed to compare isolates from MEF with isolates from NPS and to analyze the *Streptococcus pneumoniae* serotype distribution. We analyzed MEF and NPS samples in children 2 months to 5 years for vaccination status, frequency of bacterial strains, serotype/*emm*-type distribution of *S. pneumoniae, Haemophilus influenzae*, and *Streptococcus pyogenes*; and intraindividual correlation between MEF and NPS. From 2008 to 2014, MEF samples were collected from 2,138 subjects of which 2,001 (93.6%) also provided an NPS sample. In 851 of 2,138 MEF samples (39.8%), we identified organisms with confirmed pathogenic potential—*S. pyogenes*: 315 (14.7%), *S. pneumoniae*: 170 (8.0%), *Staphylococcus aureus*: 168 (7.9%), *H. influenzae*: 133 (6.2%), and *Moraxella catarrhalis*. Among NPS samples, 1,018 (50.9%) contained *S. pneumoniae*, 775 (38.7%) *H. influenzae*, 648 (32.4%) *M. catarrhalis*, and 344 (17.2%) *S. pyogenes*. Over the seven study years, the number of AOM patients steadily decreased, while the recruiting base remained constant. *S. pneumoniae* MEF isolates decreased by 86%, with serotype 3 being the most prevalent (25.7–42.9%). PCV13-non-PCV7-non-3 serotypes reduced to 0%. Among NPS, PCV7 serotypes decreased from 14.1 to 3.7%, PCV10: 17.6 to 3.7%, and PCV13: 55.3 to 25.7%. PCV13-non-PCV7-non-3 serotypes increased in the first 3 years of the study (17.1–22.9%), then decreased to 4.6% in year 7. Non-typeable *H. influenzae* reduced from 87.1 to 41.7% in MEF and from 91.4 to 54.2% in NPS. MEF and NPS isolates from the same subject were identical for 91.9% of *S. pneumoniae*, 99.0% of *S. pyogenes*, and 83.3% of *H. influenzae*. Among PCV7-vaccinated children, 5.6% had a PCV7 vaccine type in the MEF sample, and among PCV13-vaccinated children, 51.7% had a PCV13 serotype. Among non-vaccinated children, the percentages were 14.8 and 70.4. Pneumococcal conjugate vaccination has impacted the prevalence and etiology of spontaneously ruptured otitis media among children in Germany. Overall case numbers and pneumococcal vaccine type cases have strongly decreased.

## Introduction

Acute otitis media (AOM) is a painful inflammation of the middle ear with a multifactorial pathogenesis. It is usually based on an ascending, either existent or precedent, infection of the upper respiratory system via the Eustachian tube. It is assumed that in many cases a primary viral infection of the upper respiratory tract is followed by bacteria colonizing the nasopharynx immigrating via the Eustachian tube to the middle ear, which results in an infection. Due to swelling of the tube, drainage is reduced and the tympanum might perforate spontaneously (1). AOM is the number one reason for pediatric consultations and antibiotic prescriptions in industrialized countries (2).

Results of previous studies on the etiology of AOM showed that *Streptococcus pneumoniae, Haemophilus influenzae, Streptococcus pyogenes*, and *Moraxella catarrhalis* were the major bacterial pathogens involved (3, 4). For two of these pathogens, *H. influenzae* type b and *S. pneumoniae*, vaccines are available. The vaccination against *H. influenzae* type b has been recommended in Germany for children from the age of 2 months to 5 years since 1990.

*Streptococcus pneumoniae* infections are among the leading causes of morbidity and mortality among infants and children worldwide (5). *S. pneumoniae* may cause invasive pneumococcal disease (IPD), such as sepsis and meningitis, as well as non-invasive pneumococcal disease (NIPD), like otitis media, sinusitis, bronchitis, and non-bacteremic pneumonia. Irrespective of being less severe, NIPDs have a great impact on public health: nearly every child has experienced an episode of AOM by the age of 2 (6).

In Germany, pneumococcal conjugate vaccination was first recommended for high-risk children in 2001. A general recommendation for pneumococcal conjugate vaccination for all children under 2 years of age was issued in July 2006 (7) with the potential not only to be effective against invasive pneumococcal disease but also to have an impact on AOM and bacterial colonization. Initially, a seven-valent pneumococcal conjugate vaccine (PCV7, serotypes 4, 6B, 9V, 14, 18C, 19F, and 23F) was used. In 2009, higher-valent PCVs (PCV10 covering additional serotypes 1, 5, and 7F and PCV13 covering additional serotypes 1, 3, 5, 6A, 7F, and 19A) became available (8, 9). All pneumococcal conjugate vaccines (PCVs) included the five most frequent pneumococcal serotypes associated with AOM, i.e., 6B, 9V, 14, 19F, and 23F (10, 11).

Several clinical trials in other countries have demonstrated a significant reduction of AOM in PCV7-immunized children. Furthermore, the overall incidence of pneumococcal disease was reduced in PCV7-immunized children, as well as the occurrence of vaccine-serotype-associated infections. In contrast, non-vaccine-serotype-associated infections were slightly elevated (12–15), a phenomenon first reported in countries which implemented PCV7 national immunization programs (16).

This study examines the bacteriologic spectrum of middle ear fluid (MEF) and nasopharyngeal swabs (NPS) of children with spontaneously perforated AOM, providing surveillance data on pneumococcal disease and carriage in the era of routine pneumococcal vaccination in Germany. Analysis of AOM disease burden and *S. pneumoniae* serotype distribution over the study period will provide insight on the impact of ongoing childhood pneumococcal conjugate vaccination. Furthermore, a comparison of isolates from MEF with isolates from NPS will provide insight on the relationship between carriage and AOM. A report on the first 3 years of this study was previously published (17).

## Materials and Methods

### Study Design

This longitudinal, multicenter, epidemiological cross-sectional study included children 2 months to 5 years of age with acute, spontaneously draining otitis media. Since aspiration of MEF for study purposes is considered to be unethical in Germany, only cases of spontaneously draining AOM could be included. NPS were taken when parental consent was obtained. The immunization status of each subject was documented.

Our analysis encompasses the following: (1) description of the study population (demographic data, environmental influences, relevant concomitant diseases, and immunization status); (2) analysis of reported AOM case numbers from a constant recruiting base; (3) analysis of frequencies and distribution of bacterial species in MEF and NPS samples; (4) analysis of serotype/*emm*-type distribution of *S. pneumoniae, H. influenzae*, and *S. pyogenes* found in MEF and NPS samples; and (5) intraindividual correlation between *S. pneumoniae, H. influenzae*, and *S. pyogenes* serotype/*emm*-type distribution in MEF and NPS samples.

Sample size determination was based on the assumption that ~10% of the MEF samples from subjects with AOM would be positive for *S. pneumoniae*. At the start of the study in October 2008, six to eight cases of spontaneously draining AOM were seen per year in a pediatrician's office; therefore, 50 study sites were considered necessary in order to obtain 1,000 samples of MEF from subjects with spontaneously draining AOM within a 36-month enrollment period. In September 2011, the study was extended for four more years. Due to constantly decreasing numbers of children presenting with AOM, 35 more study sites were initiated, 10 of which already started recruiting within the last month of the third study year, resulting in 15 recruited subjects. At the end of the study, 43 of the 50 initial study sites had actively recruited; of the 35 additional sites, 32 had actively recruited.

This final report is based on the evaluation of all subjects included during the seven study years from 2008 to 2015, with each study year running from October 15 in 1 year to October 14 in the following year (see Supplemental Material 1 for details on participating centers).

At the first visit, demographic data including environmental parameters, relevant antecedent diseases, and immunization status were documented in a questionnaire. The pediatrician obtained MEF by ear swab sampling. If possible (depending on the child's condition and parental consent) an NPS sample was taken as well. The development and status of the AOM was evaluated at visit 2, at maximally 14 days after the inclusion visit. According to clinical routine, tympanometry or audiometry was performed (depending on the child's age), to confirm that the AOM had resolved. If the AOM was not resolved at visit 2, a follow-up visit (~30 days after the inclusion visit) was scheduled.

To exclude seasonal influences, the study was performed throughout the year. For all participants, written informed consent from children's legal guardians was obtained prior to the sampling, following the human experimentation guidelines of Good Clinical Practice, the German Drug Act, and the Declaration of Helsinki/Hong Kong. Approval was obtained from the local ethics committee of the University of Münster, Germany (0887X1-4454).

### Selection of Study Population

Each subject was included only once in this study. Inclusion criteria were as follows: age 2 months to 5 years (2–60 months), active, and spontaneously draining AOM for a maximum of 2 days. Exclusion criteria were as follows: previous ear surgery, chronic otitis media with effusion for at least 3 days, any severe chronic diseases and/or immunodeficient conditions, use of systemic steroids or other immunosuppressants, craniofacial malformations, treatment with antibiotics over a timeframe of more than 48 h prior to inclusion in this study, relevant hemorrhagic diseases, and participation in other interventional studies or clinical trials.

### Bacterial Growth Conditions

Primary material (MEF and NPS) was inoculated on the following media: Columbia blood agar, McConkey agar, chocolate agar, and Sabouraud agar. Identification of bacterial isolates was performed using routine microbiology techniques, including MALDI-TOF-MS (Bruker Biotyper, Germany), as well as bile solubility and optochin sensitivity in case of pneumococcal isolates.

### Serotyping

Serotyping of *S. pneumoniae* isolates was performed using the Neufeld-Quellung reaction, with type and factor sera provided by Statens Serum Institut, Copenhagen, Denmark. *H. influenzae* was serotyped using Difco *Haemophilus influenzae* antisera from Becton Dickinson, USA. The *emm* typing of *S. pyogenes* was performed as follows: PCR for amplification of the *emm* gene was carried out using the “all M” primers described by Podbielski et al. (18). PCR products were purified and sequenced using an automated ABI Prism 310 DNA sequencer (Applied Biosystems, Weiterstadt, Germany). The nucleotide sequences encoding the N-terminal hypervariable region of the M protein were compared with the sequences published on the website of the Centers for Disease Control and Prevention (https://www2.cdc.gov/vaccines/biotech/strepblast.asp). In case of a high degree of identity (≥95%) with a known *emm* sequence, the generated nucleotide sequence was assigned to the corresponding *emm*-type designation.

### Statistical Analysis

Univariate models using Firth's bias-reduced logistic regression (19) were constructed for each major pathogenic organism identified to cause AOM as the outcome variable, and year of infection, patient's daycare attendance, siblings' daycare attendance, smokers in the household, number of siblings in the household, patient's age, vaccination status, and nasopharyngeal carriage of the major pathogens as possible predictor variables. Univariate models with *p* < 0.2 were considered for multivariable analysis. Multivariate analysis was conducted with forward stepwise variable selection, using the Akaike information criterion to optimize model fit. Due to some small sample sizes, Fisher's exact test was used to assess changes in proportions. Variables were considered to be associated when odds ratios did not cross 1. Analyses were conducted with R (R 3.6.1, R Foundation for Statistical Computing, Vienna, Austria).

## Results

**Figure 1:**
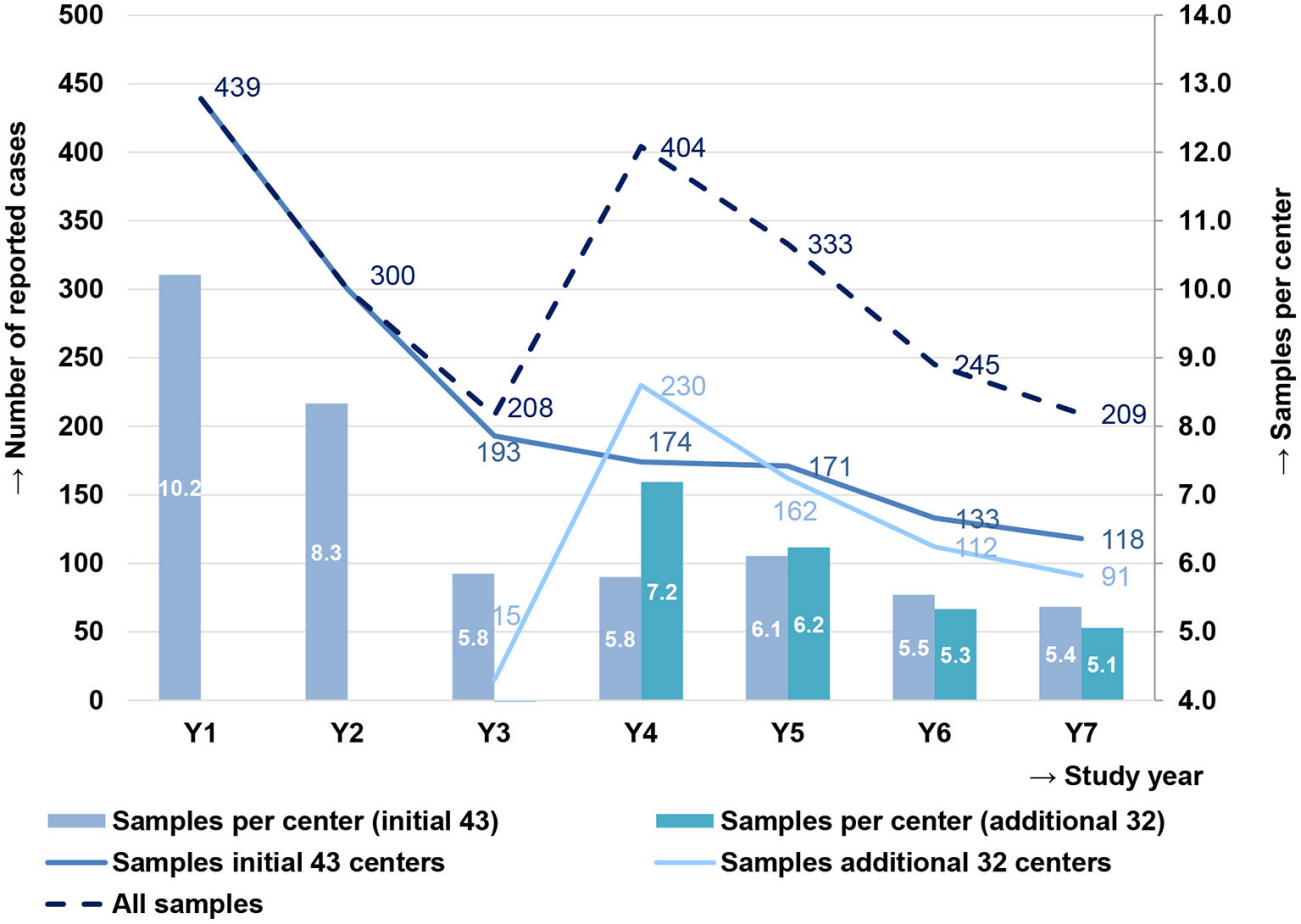
Cases of children with severe spontaneously ruptured acute otitis media (AOM) in Germany, reported by the initial 43 centers (2008–2014) and the additional 32 centers (2010–2014), and the average number of samples per initial 43 centers and additional 32 centers in each study year. Samples per center for the additional 32 centers are not shown in year 3, as 10 additional centers had started enrolling in the last month of that study year, reporting 15 cases.

During the 7 years of the study, a total of 2,268 subjects were recruited. Of those, 2,138 were included after application of the inclusion/exclusion criteria and MEF samples were obtained. For 2,001 subjects (93.6%), an NPS sample was obtained (Table 1). Over the seven study years, 439, 300, 208, 404, 333, 245, and 209 subjects were included. In the first 3 years, the number of patients steadily reduced, even though the recruiting base remained the same. Therefore, the number of recruiting centers was increased from 43 to 75 for study years 4 to 7. However, during these last 4 years, the steady decrease in recruited patients continued. The average number of cases reported per center decreased over the entire study period from 10.2 in year 1 to 5.4 in year 7, for the initial 43 centers, and from 7.2 in year 4 to 5.1 in year 7 for the additional 32 centers (Figure 1).

**Table 1:** Numbers of MEF and NPS samples from patients with severe spontaneously ruptured AOM in Germany per study year.

	**Middle ear fluid (MEF) samples**			**Nasopharyngeal swab (NPS) samples**			
	**Patients excluded (*n* =)**	**Patients included (*n* =)**	**All**	**Patients excluded (*n* =)**	**Patients included (*n* =)**	**All**	**Patients with carriage sample (%)**
Year 1	29	439	468	19	381	400	86.8
Year 2	15	300	315	13	285	298	95.0
Year 3	6	208	214	3	192	195	92.3
Year 4	35	404	439	27	389	416	96.3
Year 5	23	333	356	18	323	341	97.0
Year 6	15	245	260	14	235	249	95.9
Year 7	7	209	216	3	196	199	93.8
Total	130	2,138	2,268	97	2,001	2,098	93.6

### Demographics

The age of the included subjects varied between 2 and 60 months. The average age was 29.8 months (median 28 months). Male subjects slightly outnumbered females (1,125, 52.6%). Six hundred and nineteen (29.0%) children had no siblings, 997 (46.6%) had one sibling, and 484 (22.6%) had two or more siblings. For 38 children (1.8%), data on siblings were not obtained. One thousand two hundred and sixty-five (59.2%) of the children attended daycare, and 400 (18.7%) children were regularly exposed to cigarette smoke in the home.

### Middle Ear Fluid Isolates

Of the 2,138 MEF samples, 240 (11.2%) showed no bacterial growth, 1,059 (49.5%) presented physiological outer-ear flora (*n* = 914) or temporary contamination of the auditory canal (*n* = 145), and 26 (1.2%) had organisms of questionable pathogenic potential. In 851 (39.8%) samples, we found bacterial organisms with described pathogenic potential in AOM. Of these, 796 (37.2%) contained *S. pneumoniae, H. influenzae, S. pyogenes, M. catarrhalis*, or *S. aureus*, which are the focus of this study. Fifty-five samples (2.6%) contained *Escherichia coli, Klebsiella pneumoniae, Proteus mirabilis, Pseudomonas aeruginosa*, and *Serratia marcescens*, pathogens reported to be associated with AOM, which were not the focus of this study. A list of all MEF isolates is presented in Supplemental Material 2. Among the 796 patients with relevant pathogens identified in their MEF swabs, 758 subjects (95.2%) had a single bacterial strain isolated; for 38 (4.8%), two relevant strains were obtained.

*Streptococcus pyogenes* was recovered in 315 MEF swabs (14.7% of all 2,138 MEF samples, 39.6% of 796 MEF samples with isolates which were the focus of this study), *S. pneumoniae* in 170 (8.0%, 21.4%), *S. aureus* in 168 (7.9%, 21.1%), *H. influenzae* in 133 (6.2%, 16.7%), and *M. catarrhalis* in 10 (0.5%, 1.3%) samples (Table 2). For 37 patients, two relevant strains were isolated (*S. pyogenes*/*S. aureus*: *n* = 10; *S. pneumoniae*/*H. influenzae*: *n* = 9; *S. pyogenes*/*H. influenzae*: *n* = 6; *S. pneumoniae*/*S. aureus*: *n* = 5; *H. influenzae*/*S. aureus, M. catarrhalis*/*S. aureus*: *n* = 2 each; *S. pneumoniae*/*S. pyogenes, S. pneumoniae*/*S. pneumoniae, S. pneumoniae*/*M. catarrhalis*: *n* = 1 each). In one patient, *S. pneumoniae* was found together with *Enterobacter cloacae*, the latter most probably being a temporary contaminant of the auditory canal.

**Table 2:** Relevant pathogens isolated from MEF from patients with severe spontaneously ruptured AOM in Germany per study year.

**Isolate**	**Y1**	**%**	**%rel**	**Y2**	**%**	**%rel**	**Y3**	**%**	**%rel**	**Y4**	**%**	**%rel**	**Y5**	**%**	**%rel**	**Y6**	**%**	**%rel**	**Y7**	**%**	**%rel**	**All**	**%**	**%rel**
*Streptococcus pyogenes*	53	12.1	31.2	34	11.3	30.9	24	11.5	36.9	75	18.6	46.0	60	18.0	50.4	36	14.7	39.1	33	15.8	42.9	315	14.7	39.6
*Streptococcus pneumoniae*	43	9.8	25.3	35	11.7	31.8	12	5.8	18.5	28	6.9	17.2	21	6.3	17.6	17	6.9	18.5	14	6.7	18.2	170	8.0	21.4
*Staphylococcus aureus*	36	8.2	21.2	23	7.7	20.9	15	7.2	23.1	33	8.2	20.2	23	6.9	19.3	20	8.2	21.7	18	8.6	23.4	168	7.9	21.1
*Haemophilus influenzae*	31	7.1	18.2	17	5.7	15.5	14	6.7	21.5	26	6.4	16.0	15	4.5	12.6	18	7.3	19.6	12	5.7	15.6	133	6.2	16.7
*Moraxella catarrhalis*	7	1.6	4.1	1	0.3	0.9	0	0.0	0.0	1	0.2	0.6	0	0.0	0.0	1	0.4	1.1	0	0.0	0.0	10	0.5	1.3
Patients with relevant isolates (*n* =)	170		100.0	110		100.0	65		100.0	163		100.0	119		100.0	92		100.0	77		100.0	796		100.0
All patients included (*n* =)	439	100.0		300	100.0		208	100.0		404	100.0		333	100.0		245	100.0		209	100.0		2,138	100.0	

*%, percentage of all included patients; %rel, percentage of patients with relevant MEF isolates*.

### Nasopharyngeal Swab Isolates

**Figure 2:**
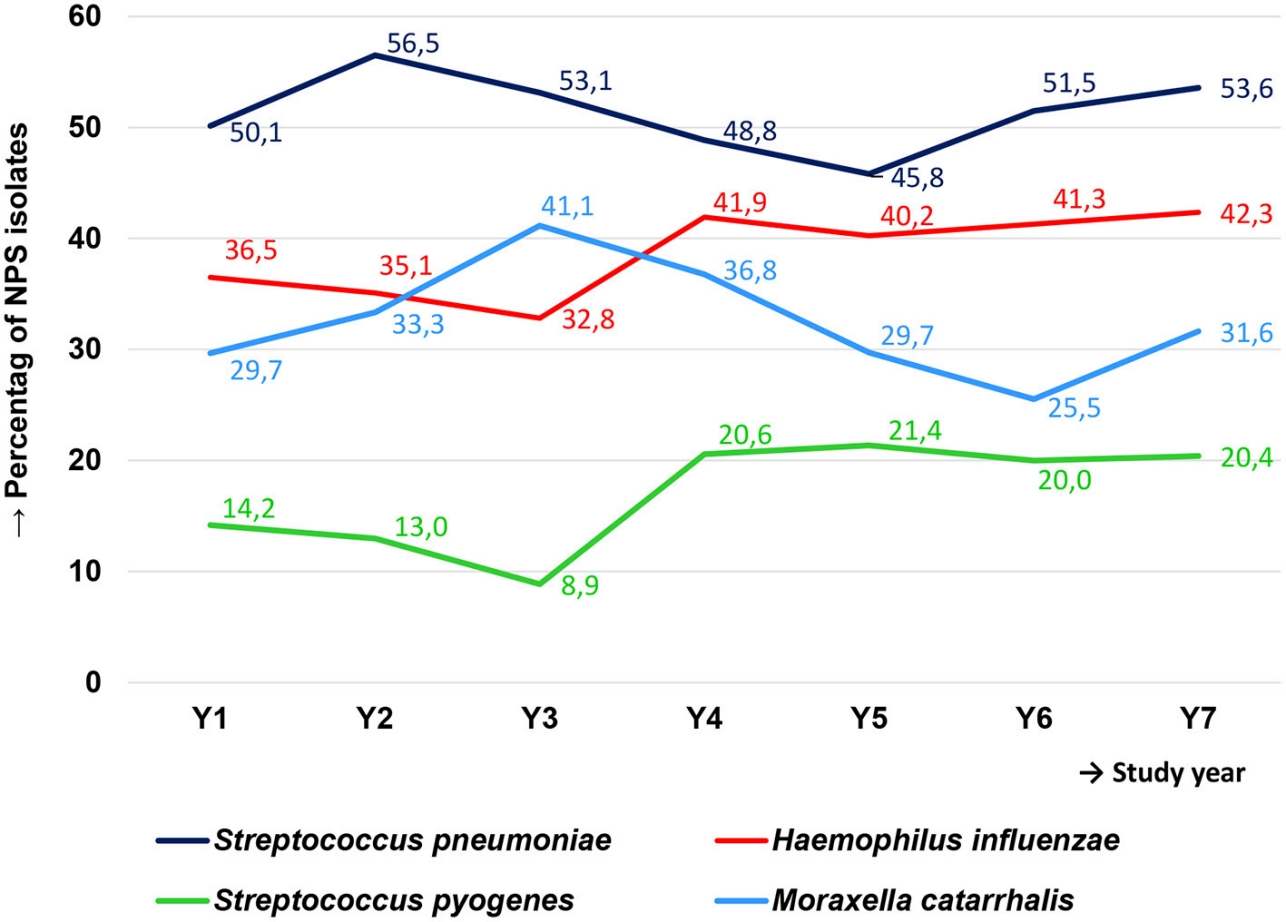
Percentage of nasopharyngeal carriage of four relevant bacterial species among children in Germany with severe spontaneously ruptured AOM over the 7-year study period.

An NPS was obtained for 2,001 (93.6%) subjects. The isolates were analyzed for the presence of the following bacteria: *S. pneumoniae, S. pyogenes, H. influenzae*, and *M. catarrhalis*. Of the 2,001 patients, 1,018 (50.9%) carried *S. pneumoniae*, 775 (38.7%) *H. influenzae*, 648 (32.4%) *M. catarrhalis*, 344 (17.2%) *S. pyogenes*, and 309 (15.4%) carried none of the abovementioned species. Eleven (0.5%) NPS swabs could not be analyzed due to technical errors in the processing in the participating centers. The presented percentages do not add up to 100, because of multiple carriage (Table 3). Over the seven study years, carriage of *S. pneumoniae* increased from 50.1 to 56.5% in year 2, continuously decreased to 45.8% in year 5, and then increased again to 53.6% in year 7. Carriage of *H. influenzae* increased from 36.5% in year 1 to 42.3% in year 7. *M. catarrhalis* carriage percentage fluctuated strongly over the seven study years between 41.4% in year 3 and 25.5% in year 6. *S. pyogenes* carriage was around 10% in years 1–3 but increased to around 20% in years 4–7 (Figure 2 and Table 3).

**Table 3:** Numbers of patients with relevant bacteria in NPS.

**Patients with**	**Y1**	**%**	**Y2**	**%**	**Y3**	**%**	**Y4**	**%**	**Y5**	**%**	**Y6**	**%**	**Y7**	**%**	**All**	**%**
NPS	381	100.0	285	100.0	192	100.0	389	100.0	323	100.0	235	100.0	196	100.0	2,001	100.0
*Streptococcus pneumoniae*	191	50.1	161	56.5	102	53.1	190	48.8	148	45.8	121	51.5	105	53.6	1,018	50.9
*Haemophilus influenzae*	139	36.5	100	35.1	63	32.8	163	41.9	130	40.2	97	41.3	83	42.3	775	38.7
*Streptococcus pyogenes*	54	14.2	37	13.0	17	8.9	80	20.6	69	21.4	47	20.0	40	20.4	344	17.2
*Moraxella catarrhalis*	113	29.7	95	33.3	79	41.1	143	36.8	96	29.7	60	25.5	62	31.6	648	32.4
Isolate lost in transport	2	0.5	0	0.0	0	0.0	0	0.0	0	0.0	0	0.0	0	0.0	2	0.1
Technical error in swab handling	6	1.6	1	0.4	0	0.0	2	0.5	0	0.0	0	0.0	0	0.0	9	0.4
No relevant bacterial growth	52	13.6	46	16.1	34	17.7	53	13.6	50	15.5	38	16.2	36	18.4	309	15.4

*Percentages do not add up to 100, because multiple carriage was observed*.

### S. pneumoniae

**Figure 3:**
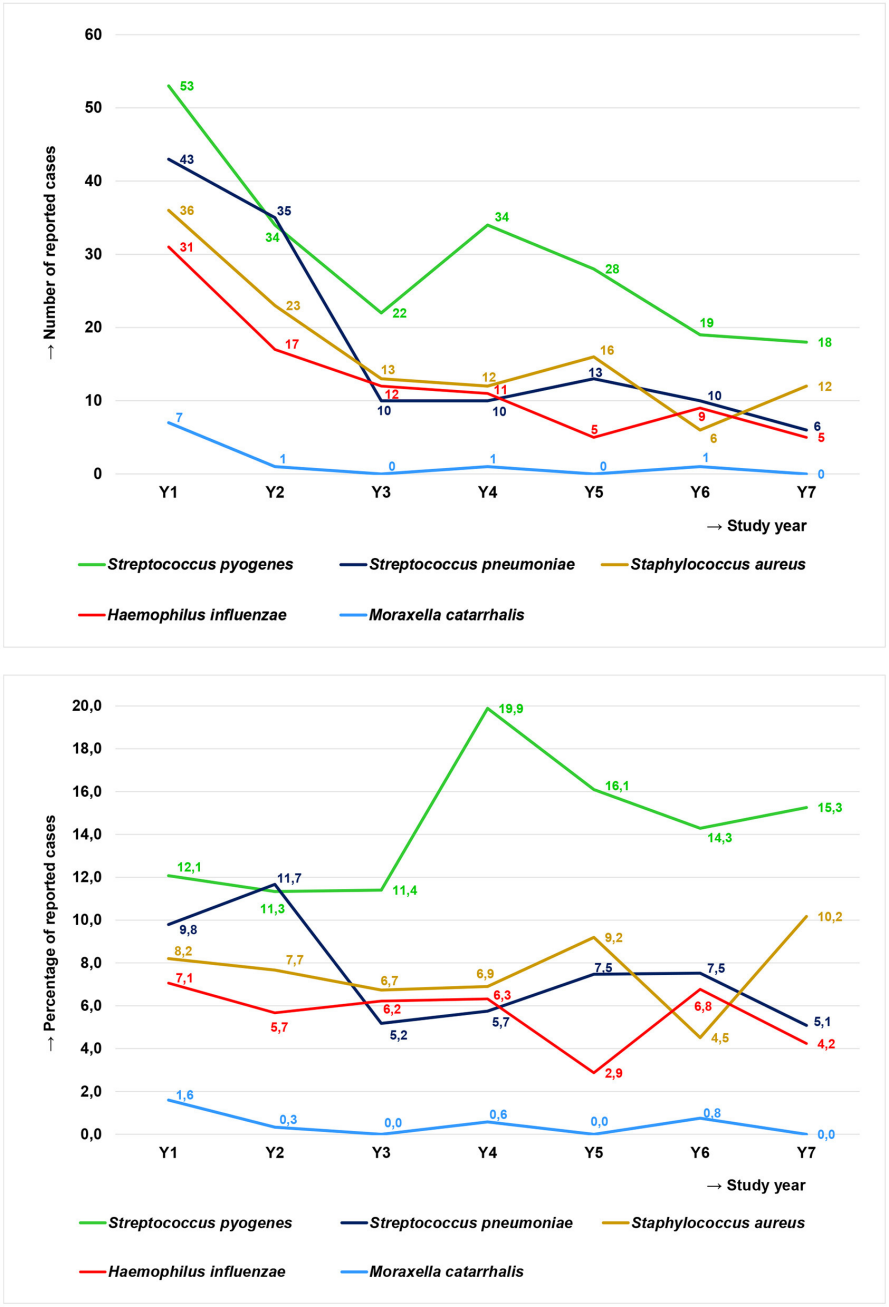
Relevant pathogens in MEF of children in Germany with severe spontaneously ruptured AOM over the seven study years reported from the initial 43 study centers depicted as reported case numbers **(Top)** and as the percentage of total reported cases **(Bottom)**.

The percentage of patients with *S. pneumoniae* isolated from MEF was 9.8% in year 1 and 11.7% in year 2. In years 3–7, this percentage decreased to 5.8–6.9 (Figure 3 and Table 2). In parallel, the number of AOM cases reported by all centers decreased from 439 samples in the first year to 209 samples in the last year of the study (Figure 1). However, regarding the initial 43 study centers, the number of AOM cases reported decreased from 439 samples in the first year to 118 samples in the last year of the study (Figure 1). *S. pneumoniae* isolates reported by these 43 centers decreased from 43 in year 1 to six in year 7 (−86%, Figure 3). For the 32 study centers added in year 4 of the study, a decrease from 230 MEF samples in year 4 to 91 MEF samples in year 7 was observed. *S. pneumoniae* isolates reported by these centers decreased from 18 in year 4 to eight in year 7 (−56%). As centers were audited for their reporting behavior, these numbers reflect a real reduction.

**Figure 4:**
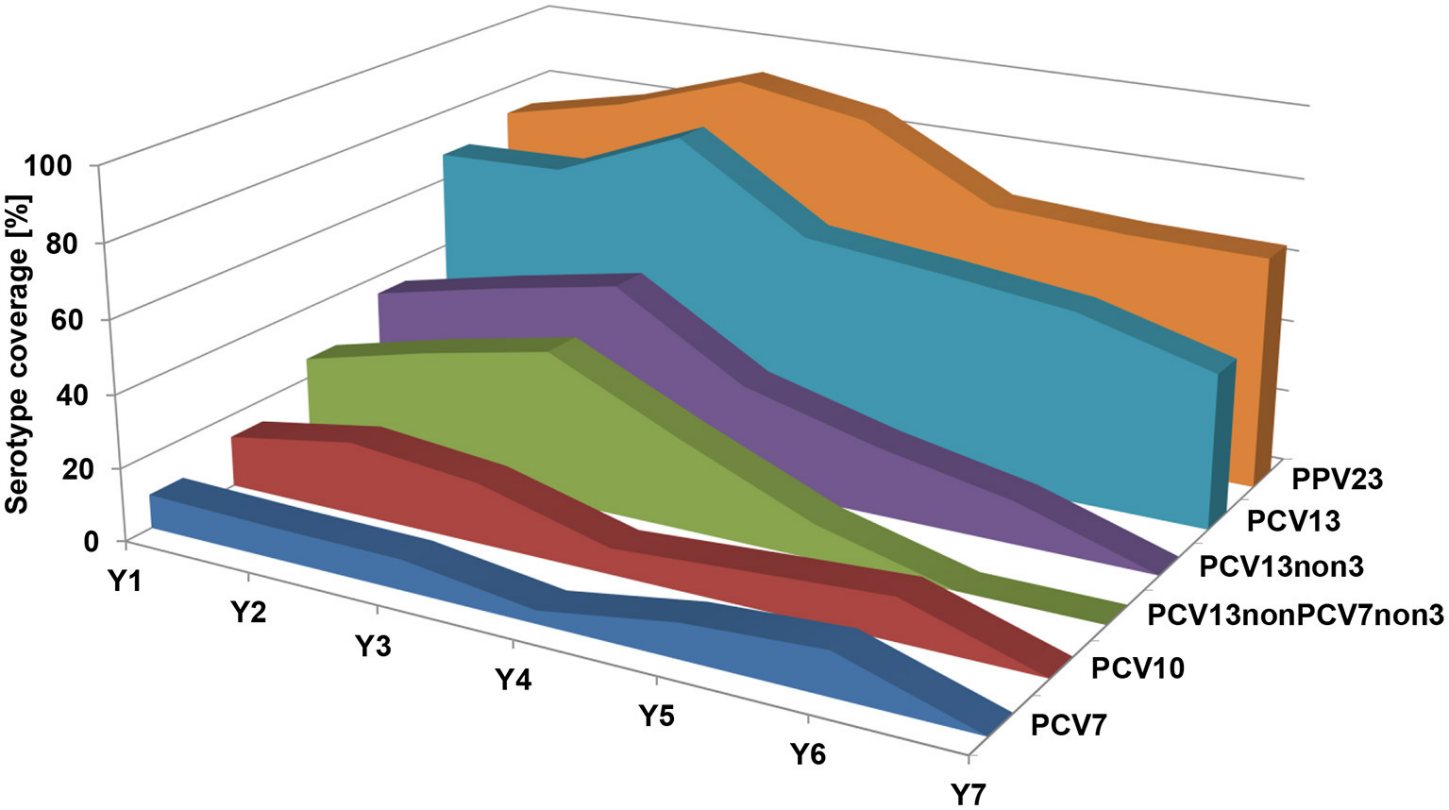
Prevalence of *S. pneumoniae* serotypes among MEF isolates over the seven study years, grouped by different vaccine formulations. PCV7, serotypes included in PCV7; PCV10, serotypes included in PCV10; PCV13, serotypes included in PCV13; PCV13-non-PCV7-non-3, PCV13 serotypes, excluding PCV7 serotypes and serotype 3; PCV13-non-3, PCV13 serotypes excluding serotype 3; PPV23, serotypes included in PPV23.

The most prevalent serotype in all seven study years was serotype 3, with a prevalence varying from 25.7% in year 2 to 42.9% in year 7. In the first three study years (2008/2009–2010/2011), serotype 19A was the second most prevalent serotype, but its prevalence decreased to zero during the last four study years (2011/2012–2014/2015). Serotype 19F persisted longer and only disappeared in the last year of the study. PCV7 and PCV10 serotypes both decreased from 9.3% and 14.0% in year 1 to 0% in year 7. PCV13-non-PCV7 serotypes, excluding serotype 3, increased from 25.6 to 41.7% in the first three study years, then decreased in the years after (25.0% in year 4; 0% in years 6 and 7). In the last study year, only six isolates with PCV13 serotypes (all serotype 3) and eight isolates with non-PCV13 serotypes were found in MEF samples (Figure 4 and Table 4).

**Table 4:** Prevalence of *S. pneumoniae* and *H. influenzae* serotypes and *S. pyogenes emm* types among MEF isolates over the seven study years.

**Serotypes *S. pneumoniae***	**Y1**	**%**	**Y2**	**%**	**Y3**	**%**	**Y4**	**%**	**Y5**	**%**	**Y6**	**%**	**Y7**	**%**	**Total**	**%**
PCV7	4	9.3	3	8.6	1	8.3	1	3.6	2	9.5	2	11.8	0	0.0	13	7.6
PCV10	6	14.0	7	20.0	2	16.7	2	7.1	2	9.5	2	11.8	0	0.0	21	12.4
PCV13	29	67.4	24	68.6	10	83.3	17	60.7	12	57.1	9	52.9	6	42.9	107	62.9
PPV23	31	72.1	28	80.0	11	91.7	24	85.7	14	66.7	11	64.7	9	64.3	128	75.3
PCV13-non-PCV7-non-3	11	25.6	12	34.3	5	41.7	7	25.0	2	9.5	0	0.0	0	0.0	37	21.8
19F	4	9.3	3	8.6	1	8.3	1	3.6	1	4.8	2	11.8			12	7.1
23F									1	4.8					1	0.6
1	2	4.7	2	5.7			1	3.6							5	2.9
7F			2	5.7	1	8.3									3	1.8
3	14	32.6	9	25.7	4	33.3	9	32.1	8	38.1	7	41.2	6	42.9	57	33.5
19A	9	20.9	8	22.9	4	33.3	6	21.4	2	9.5					29	17.1
10A							1	3.6					1	7.1	2	1.2
11A	1	2.3	2	5.7	1	8.3	5	17.9	1	4.8	1	5.9			11	6.5
12F													1	7.1	1	0.6
15B			1	2.9							1	5.9			2	1.2
22F			1	2.9					1	4.8					2	1.2
33F	1	2.3					1	3.6					1	7.1	3	1.8
6C			2	5.7											2	1.2
15A	1	2.3									1	5.9			2	1.2
15C			1	2.9			2	7.1	1	4.8	2	11.8			6	3.5
16F											1	5.9			1	0.6
21	3	7.0											1	7.1	4	2.4
23A	2	4.7	1	2.9					1	4.8					4	2.4
23B	2	4.7	1	2.9	1	8.3			1	4.8			1	7.1	6	3.5
27									1	4.8					1	0.6
28A									1	4.8					1	0.6
31	1	2.3											1	7.1	2	1.2
35B			1	2.9			1	3.6			1	5.9	1	7.1	4	2.4
35F									1				1	7.1	2	1.2
35F	1	2.3								0.0					1	0.6
38									1						1	0.6
NT			1	2.9											1	0.6
Not done	2	4.7					1	3.6			1	5.9			4	2.4
Total	43	100.0	35	100.0	12	100.0	28	100.0	21	100.0	17	100.0	14	100.0	170	100.0
**Serotypes** ***H. influenzae***	**Y1**	**%**	**Y2**	**%**	**Y3**	**%**	**Y4**	**%**	**Y5**	**%**	**Y6**	**%**	**Y7**	**%**	**Total**	**%**
b			2	11.8	2	14.3	2	8.0	2	12.5	4	22.2	3	25.0	15	11.3
d	1	3.2							1	6.3	1	5.6			3	2.3
e											2	11.1			2	1.5
f	1	3.2	2	11.8	2	14.3	5	20.0	2	12.5	2	11.1	2	16.7	16	12.0
g													1	8.3	1	0.8
NT	27	87.1	13	76.5	10	71.4	18	72.0	11	68.8	8	44.4	5	41.7	92	69.2
Not done	2	6.5									1	5.6	1	8.3	4	3.0
Total	31	100.0	17	100.0	14	100.0	25	100.0	16	100.0	18	100.0	12	100.0	133	100.0
***emm*** **types** ***S. pyogenes***	**Y1**	**%**	**Y2**	**%**	**Y3**	**%**	**Y4**	**%**	**Y5**	**%**	**Y6**	**%**	**Y7**	**%**	**Total**	**%**
1	16	30.2	10	29.4	8	33.3	22	29.3	17	28.3	7	19.4	16	48.5	96	30.5
2	2	3.8	2	5.9	1	4.2	5	6.7			1	2.8			11	3.5
3	1	1.9	2	5.9	5	20.8	1	1.3	6	10.0	6	16.7	2	6.1	23	7.3
4	2	3.8			1	4.2	3	4.0	2	3.3	1	2.8	1	3.0	10	3.2
5	1	1.9							1	1.7					2	0.6
6	3	5.7	6	17.6	1	4.2	4	5.3	3	5.0	3	8.3	1	3.0	21	6.7
9													1	3.0	1	0.3
12	6	11.3	5	14.7	2	8.3	16	21.3	15	25.0	4	11.1	3	9.1	51	16.2
22							1	1.3							1	0.3
28	3	5.7	3	8.8	3	12.5	14	18.7	4	6.7	3	8.3	2	6.1	32	10.2
29	1	1.9		0.0			1	1.3			1	2.8			3	1.0
44							1	1.3	1	1.7	1	2.8			3	1.0
48													1	3.0	1	0.3
75	6	11.3			1	4.2	5	6.7	4	6.7	1	2.8	2	6.1	19	6.0
77	2	3.8	1	2.9											3	1.0
87	1	1.9													1	0.3
89	6	11.3	4	11.8	2	8.3	2	2.7	7	11.7	4	11.1	2	6.1	27	8.6
118													1	3.0	1	0.3
Not done	3	5.7	1	2.9							4	11.1	1	3.0	9	2.9
Total	53	100.0	34	100.0	24	100.0	75	100.0	60	100.0	36	100.0	33	100.0	315	100.0

*Orange, PCV7 serotypes; green, +PCV10; blue, +PCV13; yellow, +PPV23 (6A not included in PPV23)*.

**Figure 5:**
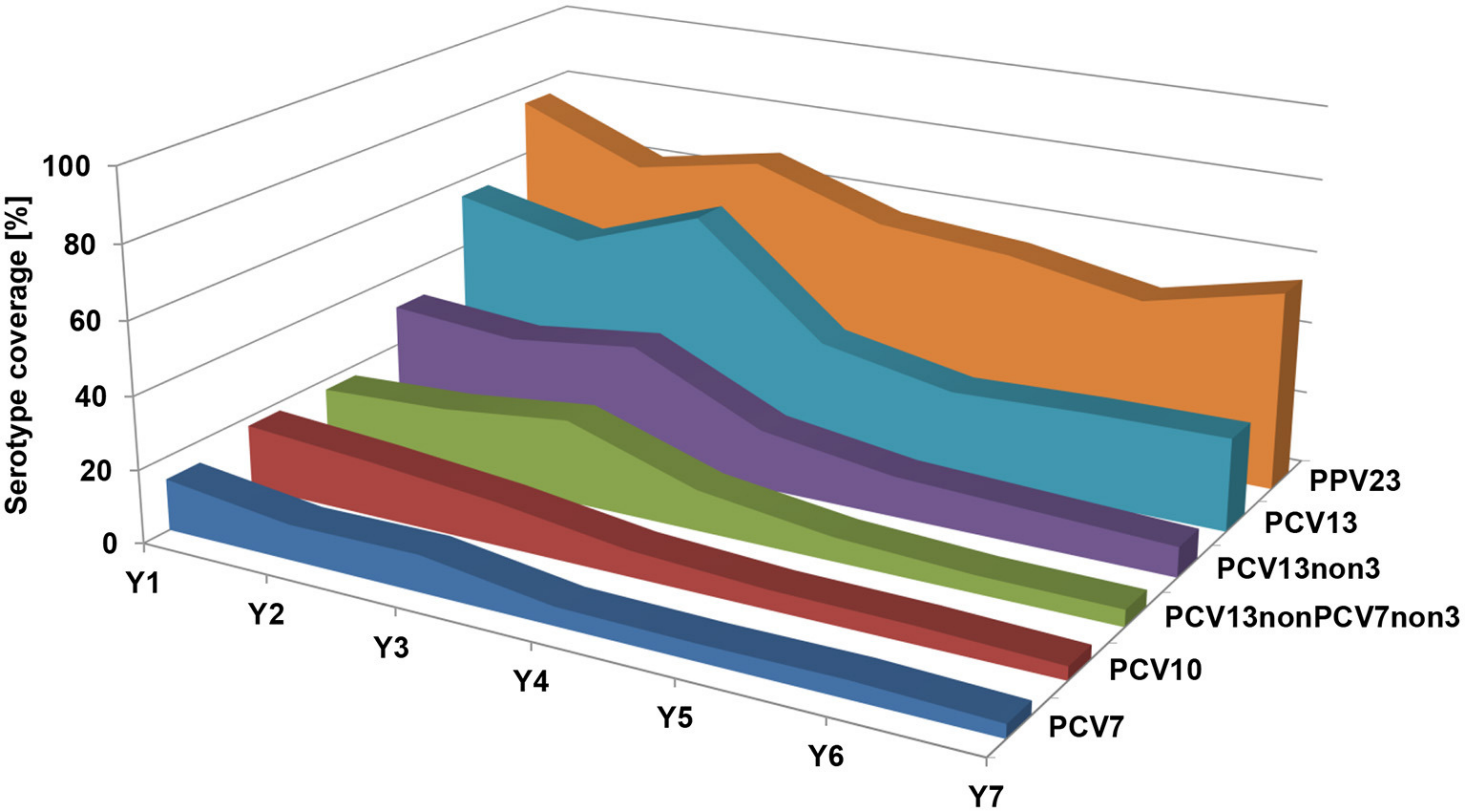
Prevalence of *S. pneumoniae* serotypes among NPS isolates over the seven study years, grouped by different vaccine formulations. PCV7, serotypes included in PCV7; PCV10, serotypes included in PCV10; PCV13, serotypes included in PCV13; PCV13-non-PCV7-non-3, PCV13 serotypes, excluding PCV7 serotypes and serotype 3; PCV13-non-3, PCV13 serotypes excluding serotype 3; PPV23, serotypes included in PPV23.

The percentage of subjects with *S. pneumoniae*-positive NPS varied between 56.5% in year 2 and 45.8% in year 5, not showing a directed trend (Table 3). However, the serotype distribution of these isolates changed dramatically over the seven study years. The proportion of PCV7 serotypes in *S. pneumoniae*-positive NPS samples decreased from 14.1% in year 1 to 3.7% in year 7, PCV10: 17.6 to 3.7%, and PCV13: 55.3 to 25.7%. PCV13-non-PCV7-non-serotype 3 increased in the first 3 years of the study (17.1–22.9%), but decreased to 4.6% in year 7. Serotypes 11A, 15A/B/C, 23A, and 23B were the most prevalent non-PCV13 serotypes in year 7 (Figure 5 and Table 5).

**Table 5:** Prevalence of *S. pneumoniae* and *H. influenzae* serotypes and *S. pyogenes emm* types among NPS isolates over the seven study years.

**Serotypes *S. pneumoniae***	**Y1**	**%**	**Y2**	**%**	**Y3**	**%**	**Y4**	**%**	**Y5**	**%**	**Y6**	**%**	**Y7**	**%**	**Total**	**%**
PCV7	28	14.1	16	9.7	11	10.1	10	5.1	7	4.6	6	4.9	4	3.7	82	7.8
PCV10	35	17.6	25	15.2	13	11.9	14	7.2	8	5.3	6	4.9	4	3.7	105	10.0
PCV13	110	55.3	79	47.9	66	60.6	60	30.8	36	23.8	31	25.2	28	25.7	410	39.0
PPV23	150	75.4	101	61.2	74	67.9	109	55.9	80	53.0	57	46.3	60	55.0	631	60.0
PCV13-non-PCV7-non-3	34	17.1	31	18.8	25	22.9	22	11.3	10	6.6	6	4.9	5	4.6	133	12.7
4					1	0.9									1	0.1
6B	5	2.5	2	1.2	1	0.9	2	1.0							10	1.0
9V	1	0.5	2	1.2											3	0.3
14			1	0.6	1	0.9									2	0.2
18C	1		1	0.6					1						3	0.3
19F	15	7.5	9	5.5	7	6.4	8	4.1	5	3.3	6	4.9	4	3.7	54	5.1
23F	6	3.0	1	0.6	1	0.9			1	0.7					9	0.9
1	5	2.5	3	1.8			2	1.0	1	0.7					11	1.0
5			1	0.6											1	0.1
7F	2	1.0	5	3.0	2	1.8	2	1.0							11	1.0
3	48	24.1	32	19.4	30	27.5	28	14.4	19	12.6	19	15.4	19	17.4	195	18.6
6A	6	3.0	5	3.0	5	4.6	2	1.0							18	1.7
19A	21	10.6	17	10.3	18	16.5	16	8.2	9	6.0	6	4.9	5	4.6	92	8.8
8							1	0.5			1	0.8			2	0.2
9N	2	1.0	1	0.6			1	0.5			1	0.8	1	0.9	6	0.6
10A	4	2.0	4	2.4	1	0.9	10	5.1	11	7.3	4	3.3	5	4.6	39	3.7
11A	19	9.5	9	5.5	3	2.8	22	11.3	20	13.2	9	7.3	12	11.0	94	8.9
12F							1	0.5			1	0.8	4	3.7	6	0.6
15B	10	5.0	4	2.4	4	3.7	4	2.1	4	2.6	5	4.1	3	2.8	34	3.2
17F	2	1.0	1	0.6			1	0.5			1	0.8	1	0.9	6	0.6
20							1	0.5	1	0.7					2	0.2
22F	3	1.5	4	2.4	4	3.7	5	2.6	4	2.6	3	2.4	2	1.8	25	2.4
33F	6	3.0	4	2.4	1	0.9	5	2.6	4	2.6	1	0.8	4	3.7	25	2.4
6C	2	1.0	6	3.6	2	1.8	6	3.1	5	3.3	3	2.4			24	2.3
7C							1	0.5			1	0.8			2	0.2
10B			1	0.6											1	0.1
13											1	0.8	1	0.9	2	0.2
15A	2	1.0	2	1.2			2	1.0	4	2.6	6	4.9	1	0.9	17	1.6
15C	4	2.0	4	2.4	4	3.7	7	3.6	9	6.0	6	4.9	5	4.6	39	3.7
16F	1	0.5	1	0.6			3	1.5	6	4.0	1	0.8	2	1.8	14	1.3
17A			1	0.6											1	0.1
18A			1	0.6											1	0.1
21	4	2.0	5	3.0	2	1.8	4	2.1	3	2.0	8	6.5	5	4.6	31	2.9
22A							1	0.5							1	0.1
23A	9	4.5	7	4.2	3	2.8	10	5.1	8	5.3	8	6.5	8	7.3	53	5.0
23B	3	1.5	7	4.2	4	3.7	12	6.2	11	7.3	8	6.5	6	5.5	51	4.9
24A			1	0.6											1	0.1
24B											1	0.8	1	0.9	2	0.2
24F			1	0.6	1	0.9	6	3.1	3	2.0	3	2.4	4	3.7	18	1.7
28A			1	0.6					2	1.3			1	0.9	4	0.4
28F							2	1.0							2	0.2
31	2	1.0	3	1.8	1	0.9	2	1.0	3	2.0	1	0.8	3	2.8	15	1.4
34	4	2.0	1	0.6	2	1.8	1	0.5	1	0.7	4	3.3			13	1.2
35B			4	2.4	4	3.7	7	3.6	4	2.6	5	4.1	3	2.8	27	2.6
35C	1	0.5									1	0.8	1	0.9	3	0.3
35F	7	3.5	9	5.5	2	1.8	15	7.7	7	4.6	5	4.1	5	4.6	50	4.8
37					1	0.9			1	0.7					2	0.2
38	4	2.0	2	1.2	2	1.8	4	2.1	3	2.0	3	2.4	2	1.8	20	1.9
NT			2	1.2	2	1.8	1	0.5	1	0.7	1	0.8	1	0.9	8	0.8
Total	199	100.0	165	100.0	109	100.0	195	100.0	151	100.0	123	100.0	109	100.0	1,051	100.0
**Serotypes** ***H. influenzae***	**Y1**	**%**	**Y2**	**%**	**Y3**	**%**	**Y4**	**%**	**Y5**	**%**	**Y6**	**%**	**Y7**	**%**	**Total**	**%**
a			1	1.0			1	0.6					2	2.4	4	0.5
b	6	4.3	8	7.9	5	7.7	18	11.0	16	12.3	18	17.8	14	16.9	85	10.9
c													1	1.2	1	0.1
d									3	2.3					3	0.4
e	1	0.7	1	1.0	2	3.1	7	4.3	3	2.3	3	3.0			17	2.2
f	4	2.9	15	14.9	12	18.5	33	20.2	11	8.5	7	6.9	13	15.7	95	12.1
NT	127	91.4	76	75.2	46	70.8	104	63.8	96	73.8	63	62.4	45	54.2	557	71.2
Spontaneous agglutination									1	0.8	10	9.9	8	9.6	19	2.4
Not done	1	0.7													1	0.1
Total	139	100.0	101	100.0	65	100.0	163	100.0	130	100.0	101	100.0	83	100.0	782	100.0
***emm*** **types** ***S. pyogenes***	**Y1**	**%**	**Y2**	**%**	**Y3**	**%**	**Y4**	**%**	**Y5**	**%**	**Y6**	**%**	**Y7**	**%**	**Total**	**%**
1	20	37.0	9	24.3	6	33.3	27	33.3	17	24.6	12	25.5	15	37.5	106	30.6
2	2	3.7	1	2.7	1	5.6	5	6.2	1	1.4	1	2.1	1	2.5	12	3.5
3	4	7.4	3	8.1	5	27.8	2	2.5	5	7.2	5	10.6	2	5.0	26	7.5
4							3	3.7	3	4.3	1	2.1	3	7.5	10	2.9
5									1	1.4					1	0.3
6	3	5.6	6	16.2	1	5.6	4	4.9	7	10.1	8	17.0	1	2.5	30	8.7
9			1	2.7											1	0.3
12	7	13.0	8	21.6	3	16.7	17	21.0	16	23.2	5	10.6	5	12.5	61	17.6
22							1	1.2					1	2.5	2	0.6
28	6	11.1	2	5.4			9	11.1	4	5.8	2	4.3	2	5.0	25	7.2
29	1	1.9					2	2.5							3	0.9
44							1	1.2	1	1.4	1	2.1	1	2.5	4	1.2
48													1	2.5	1	0.3
75	5	9.3			1	5.6	5	6.2	6	8.7	4	8.5	3	7.5	24	6.9
77	1	1.9	1	2.7					1	1.4					3	0.9
89	5	9.3	6	16.2	1	5.6	5	6.2	7	10.1	7	14.9	5	12.5	36	10.4
PCR negative	0										1	2.1			1	0.3
Total	54	100.0	37	100.0	18	100.0	81	100.0	69	100.0	47	100.0	40	100.0	346	100.0

*Orange, PCV7 serotypes; green, +PCV10; blue, +PCV13; yellow, +PPV23 (6A not included in PPV23)*.

### H. influenzae

*H. influenzae* was found in MEF of, in average, 6.2% of the enrolled patients (4.5–7.3%; Table 2). In the original 43 study centers, the number of *H. influenzae* decreased from 31 cases in year 1 to only five cases in year 7 (Figure 3). Non-typeable *H. influenzae* (NT-Hi) made up 87.1% of the *H. influenzae* MEF isolates in year 1, but this percentage decreased to 68.8% in year 5. In years 6 and 7, slightly over 40% of isolates were NT. Capsulated *H. influenzae*, including capsular types b, d, e, f, and g, were sporadically found in the first 5 years, but became more prevalent in the last two study years (year 1: 6.4%, year 6/year 7: 50.0%; Table 4). *H. influenzae* was found in around 40% (32.8–42.3) of NPS samples (Table 3), and in accordance to the MEF samples, most were NT-Hi. During the period of the study, a reduction in the NT-Hi proportion, from 91.4% in year 1 to 54.2% in year 7, was observed (Table 5).

### S. pyogenes

*Streptococcus pyogenes* was found in MEF of 14.7% (11.3–18.6%) of patients and among 17.2% (8.9–21.4) of NPS samples (Tables 2, 3). In the original 43 study centers, the number of *S. pyogenes*-positive cases decreased from 53 in year 1 to 18 in year 7 (Figure 3). Most common *emm* types among MEF and NPS samples were *emm*1 (MEF 30.5%, NPS 30.6%), *emm*12 (16.2%/17.6%), *emm*28 (10.2%/7.2%), *emm*89 (8.6%/10.4%), *emm*3 (7.3%/7.5%), *emm*6 (6.7%/8.7%), and *emm*75 (6.0%/6.9%), with very little variation in the distribution over the seven study years (Tables 4, 5).

### *M. catarrhalis* and *S. aureus*

*Moraxella catarrhalis* was found in MEF of, in average, 0.5% of patients (0.0–1.6%), with a total of only 10 isolates over the seven study years (Figure 3 and Table 2). Among NPS isolates, *M. catarrhalis* was found in 32.4% (25.5–41.1%) of the patients (Table 3).

*Staphylococcus aureus* was initially not among the bacterial pathogens considered as relevant in this study about severe spontaneously ruptured AOM in Germany. Therefore, NPS samples were not routinely screened for the presence of this bacterial species. Nevertheless, *S. aureus* was found in MEF of 7.9% (6.9–8.6%) of the patients (Table 2). To address the question whether the unexpectedly high percentage of *S. aureus* in MEF samples was due to contamination from skin colonization, in study years 3–4, the healthy ear of 116 consecutive patients was swabbed and tested for the presence of *S. aureus*. Of the 13 patients with *S. aureus* in MEF, seven (53.8%) had *S. aureus* in the healthy ear as well. Among 103 patients that did not have *S. aureus* in MEF, only three (2.9%) had *S. aureus* in the healthy ear.

### Comparison of MEF and NPS Isolates

A comparison of isolates from MEF and NPS obtained from the same subject showed that isolates were potentially identical for 91.9% of *S. pneumoniae* (judged by serotype, 114 out of 124 MEF–NPS sample pairs for which both were positive for *S. pneumoniae*), 99.0% of *S. pyogenes* (judged by *emm* type, 197 out of 199 MEF–NPS sample pairs, positive for *S. pyogenes*), and 83.3% of *H. influenzae* (judged by serotype, 80 out of 96 MEF–NPS sample pairs, positive for *H. influenzae*). The complete MEF and NPS isolate comparison is listed in Supplemental Material 3.

### Vaccination Status Data

**Figure 6:**
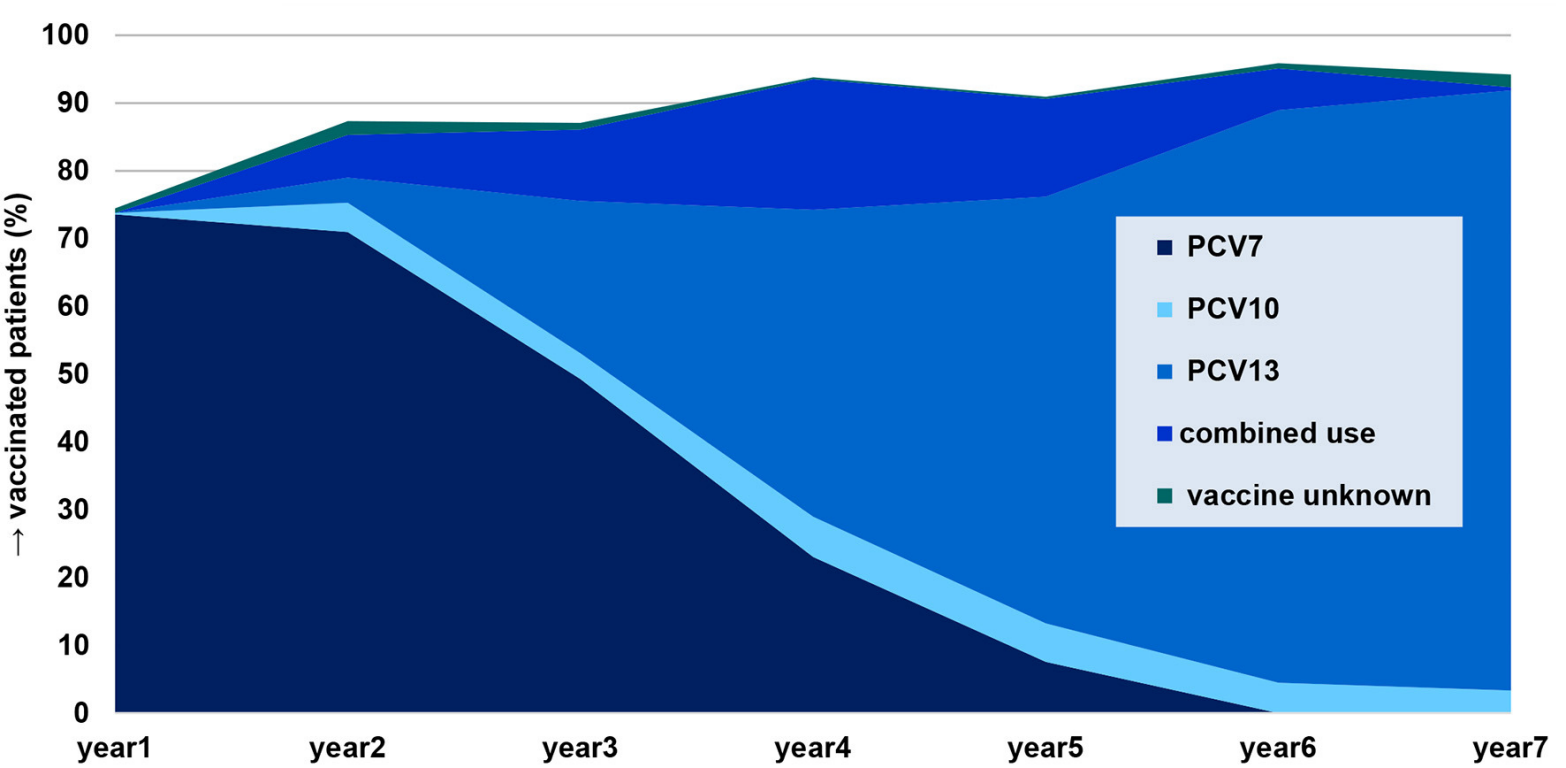
Percentage of patients with severe spontaneously ruptured AOM vaccinated with at least one dose of a pneumococcal conjugate vaccine (*n* = 1,884), categorized by vaccine type.

Of our 2,138 subjects, 1,884 (88.1%) had been vaccinated with at least one dose of a PCV [unvaccinated: 244 (11.4%), vaccination status unknown: 10 (0.5%)]. The percentage of vaccinated children increased from 74.5% in year 1 of the study to 94.3% in year 7, with a peak of 95.9% in year 6. Among the vaccinated patients, 757 (40.2%) had been vaccinated with PCV7, 842 (44.7%) with PCV13, and 83 (4.4%) with PCV10. A combination of PCVs had been received by 183 (9.7%) patients, with PCV7 followed by PCV13 being the most common combination. For 19 patients, the vaccine used could not be ascertained (Figure 6 and Supplemental Material 4).

One thousand five hundred and forty-four (82.0%) patients were vaccinated appropriately according to age [not according to age: 326 (17.3%), unknown: 14 (0.7%)]. Most patients not vaccinated appropriately according to age were lacking a booster dose (*n* = 246, 75.5%).

**Figure 7:**
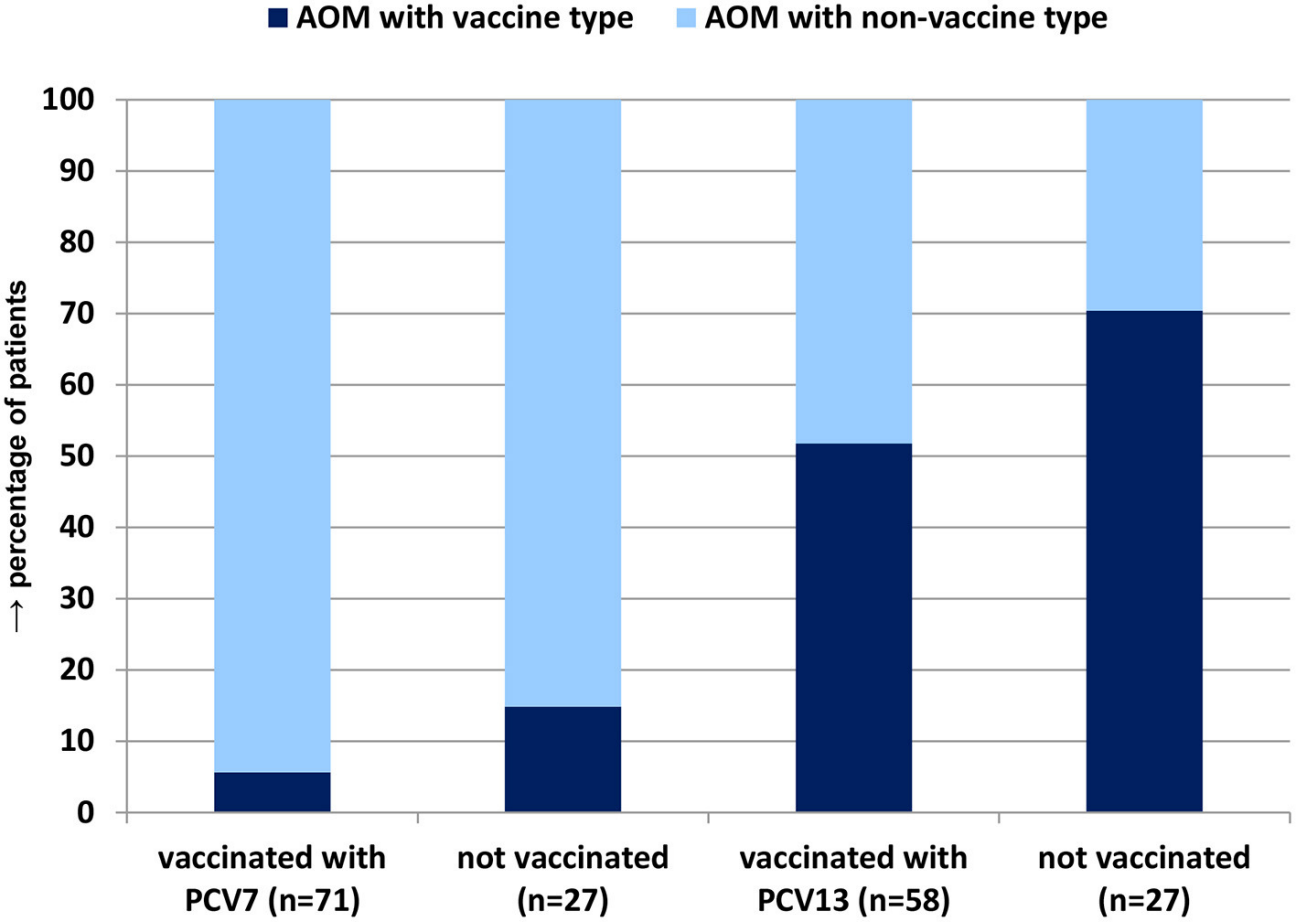
Percentage of *S. pneumoniae* vaccine type severe spontaneously ruptured AOM among patients vaccinated with PCV7 or PCV13 vs. non-vaccinated patients.

Of the 169 patients with *S. pneumoniae* isolated from MEF, 141 were vaccinated (27 not vaccinated, one unknown). Among PCV7-vaccinated children (*n* = 71), 5.6% had a PCV7 vaccine type in MEF, and among PCV13-vaccinated children (*n* = 58), 51.7% had a PCV13 serotype (PCV10, combinations, vaccine unknown: *n* = 12). Among non-vaccinated children (*n* = 27), the percentages were 14.8 and 70.4% (Figure 7).

Carriage levels of vaccinated and unvaccinated patients were not different from each other: *S. pneumoniae*: vaccinated 50.3%, non-vaccinated 55.0%; *H. influenzae*: 39.3/32.6%; *S. pyogenes*: 17.4/16.1%; and *M. catarrhalis*: 32.9/24.8%.

Among the 1,018 children positive for *S. pneumoniae* in their NPS, 892 (87.6%) were vaccinated [120 (11.8%) unvaccinated; 6 (0.6%) unknown]. Among the vaccinated children carrying *S. pneumoniae*, 6.1% had a PCV7 serotype, 7.8% PCV10, and 26.9% PCV13. Among the unvaccinated children, these percentages were 18.3, 22.5, and 38.3, respectively. Over the seven study years, carriage PCV7 serotype *S. pneumoniae* among vaccinated children increased from 7.0 to 11.2% in years 1–3, and then decreased to 3.0% in year 7, whereas it remained at around 20% over the whole study period in non-vaccinated children. Similarly, PCV13 serotype carriage increased from 33.8 to 60.7% in the first three study years and then decreased to 25.0% in year 7, but remained at around 60% among non-vaccinated children over the entire study period.

### Interspecies and Demographic Associations

The percentage of children with severe spontaneously ruptured AOM with at least one person who admitted to smoking in the home remained consistent, between 16 and 22% over the course of the study. Among children with AOM from any pathogen, 55–63% attended daycare. Between 64 and 72% of children with AOM from any cause had siblings in the household; for children with *S. pneumoniae* AOM, 53–70% of children had siblings in the household. The percentage of children with any-cause AOM who had siblings in daycare remained between 51 and 61% over the study period. For detailed information, see Supplemental Material 5.

**Figure 8:**
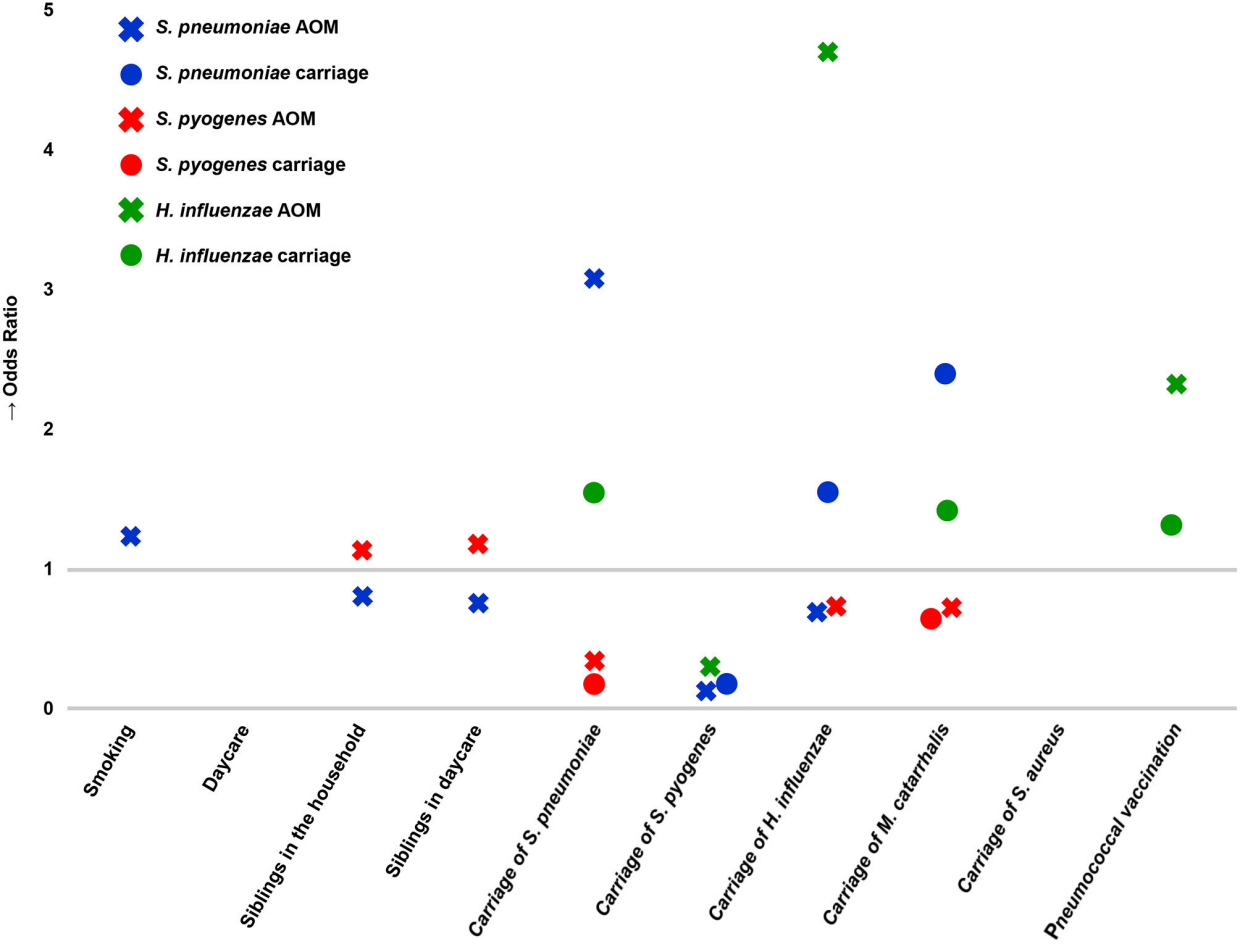
Associations of acute otitis media (crosses) and nasopharyngeal carriage (circles) with interspecies carriage and demographic factors. Associations with an odds ratio <1 indicate decreased occurrence of an event; odds ratios >1 indicate increased occurrence of an event. Associations were determined with univariate logistic regression, and results for which the 95% confidence interval of the odds ratio did not cross 1 are shown.

*Streptococcus pneumoniae* AOM cases were positively associated with carriage of *S. pneumoniae* and smoking in the household and negatively associated with carriage of *S. pyogenes*, carriage of *H. influenzae*, siblings in daycare, and siblings in the household. *S. pyogenes* AOM cases were positively associated with siblings in the household and having siblings in daycare and negatively associated with carriage of *S. pneumoniae*, carriage of *H. influenzae*, and carriage of *M. catarrhalis*. *H. influenzae* AOM cases were positively associated with carriage of *H. influenzae* and with receiving at least one PCV dose and negatively associated with carriage of *S. pyogenes*. *M. catarrhalis* AOM cases showed no associations with demographic variables or carriage of pathogens, as sample sizes were low. *S. pneumoniae* carriage was positively associated with carriage of *H. influenzae* and carriage of *M. catarrhalis* and negatively associated with carriage of *S. pyogenes*. *S. pyogenes* carriage was negatively associated with carriage of *S. pneumoniae* and with carriage of *M. catarrhalis*. *H. influenzae* carriage was positively associated with carriage of *S. pneumoniae* and carriage of *M. catarrhalis* and with receiving at least one dose of a PCV (Figure 8).

## Discussion

The German National Reference Center for Streptococci has presented only limited data on AOM of the prevaccination period in Germany (20). The current work is the first systematic analysis of MEF samples from children with severe spontaneously draining AOM in Germany. The major strength of this study is the high percentage of children for whom, together with the MEF sample, an NPS sample was obtained. Additional strengths of the study include the solid recruiting base, which supports observations of changes in case numbers, as well as a high level of completeness of the case report forms. Since investigational tympanocentesis is not allowed in Germany, only children with spontaneously draining AOM could be enrolled. This might have limited the study to the more severe cases of AOM.

The most interesting observation of this study is the enormous reduction in recruited cases over the years. This was not related to reporting fatigue of the participating centers and persisted despite the inclusion of additional centers; thus, we are confident that this reflects a real reduction in children presenting with spontaneously draining AOM. Population-level data on pediatric diagnoses in the region of North-Rhine, Germany (9.5 million inhabitants), confirm a reduction of purulent AOM from rank 7 (8% of all diagnoses) in 2008 to rank 17 (4.7% of all diagnoses) in 2014 (http://www.gbe-bund.de). A possible mechanism for this reduction is the prevention of early pneumococcal AOM episodes, as described by Dagan et al., who state that “existing PCVs can prevent early episodes of otitis media associated with vaccine serotypes, resulting in a reduction of subsequent complex cases caused by non-vaccine serotypes and other otopathogens, which contribute considerably to the disease burden” (21). Our study seems to corroborate this hypothesis, with a 73% reduction in spontaneously draining otitis media cases over 7 years for the initial 43 centers and a 60% reduction over 4 years for the additional 32 centers. This might indicate that PCV vaccination is associated with decreases in spontaneously draining otitis media, as found before in the Kaiser Permanente study in California, which reported a serotype-specific effectiveness of 66.7% for children with spontaneously draining ears, and in a study from Israel, where a 77% reduction of all-pneumococcal and a 60% reduction of all-cause otitis media incidences were observed (12, 22).

Our study found bacteria with known pathogenic potential in <40% of the cases. This probably indicates the viral origin for the remaining cases, possible misdiagnosis in very young children, or sample collection difficulties (23). Almost all cases were single-species bacterial infections confirming previous findings (24). *S. pyogenes* proved the most prevalent of the known pathogens, followed by *S. pneumoniae* and *S. aureus*, similar to other studies (25–27). *M. catarrhalis* was extremely rare, which contrasts the data from Finland and the US (24). The percentage of AOM cases from *S. pneumoniae* reduced slightly over the study duration (25.3% of relevant isolates in year 1 to 18.2% in year 7), whereas *S. pyogenes* percentages have increased (31.2% in year 1; 42.9% in year 7). Reductions of *S. pneumoniae* accompanied by an increase in *S. pyogenes* prevalence have been reported from other countries (28, 29). The other pathogens showed only minor fluctuations over the duration of the study.

NPS data showed relatively stable percentages for *S. pneumoniae* and *H. influenzae*, but an increasing trend for *S. pyogenes*, which corresponds to the increasing isolation of *S. pyogenes* from MEF. *M. catarrhalis* showed high, although fluctuating, carriage percentages. The fact that *M. catarrhalis* was frequently carried, but rarely isolated from MEF, indicates the minor role of this pathogen in severe, spontaneously ruptured otitis media (25–27). The increase of *S. pyogenes* as pathogen in severe AOM is worrisome and has been reported from several other countries (28, 29).

Our study showed a dramatic decrease (−86%) in the cases of *S. pneumoniae* AOM, reported by the initial recruiting base (43 centers) over the seven study years. We report a total disappearance of PCV7 serotypes, as well as PCV13 serotypes, except for serotype 3, which shows the impact of PCVs on AOM caused by vaccine serotypes, similar to reports from Israel (22). Apart from serotype 3, serotype 19F was the most persistent serotype, disappearing only in the last study year. Serotype 19A increased in the PCV7 period, but swiftly disappeared after the switch to higher-valent vaccination in Germany. As the vast majority of vaccinated children were vaccinated with PCV13 (>90%), this confirms the effectiveness of this formulation against AOM caused by serotype 19A, as reported in a study from the US (30). The only persisting PCV13 serotype was serotype 3, with six residual cases in year 7. However, when looking at the initial 43 recruiting centers, the number of reported serotype 3 cases reduced from 14 cases in year 1 to two in year 7. In the additional 32 recruiting centers, serotype 3 case numbers fluctuated between four in year 4, two in year 5, one in year 6, and four again in year 7. These data show that PCV13 has a limited effect on serotype 3 AOM. Case numbers of serotype 3 AOM decreased in line with the overall reduction in AOM cases reported over the course of the study, but they never disappeared, as happened with the other vaccine serotypes. Interestingly enough, only six PCV13 serotypes were detected in our MEF samples, even though all 13 were found in NPS. *S. pneumoniae* carriage percentages did not change much over the study period, but the serotype distribution showed a steep decrease of vaccine serotypes, with a 74% reduction in carriage of PCV13-non-3 serotypes and a 28% reduction in carriage of serotype 3. Non-PCV13 serotypes occurred sporadically in MEF in year 7 (eight cases, with eight different serotypes), not showing a single dominant serotype occupying the vacant niche.

With only 4–7% of MEF isolates, *H. influenzae* played a minor role in spontaneously ruptured otitis media in Germany, even though 30–40% of children carried this bacterium in their nasopharynx. Remarkably, the percentage of NT-Hi steadily decreased over the study period, both in MEF as well as in carriage, resulting in an increasing percentage of encapsulated isolates, including *H. influenzae* type b, under moderate Hib vaccination uptake in Germany (80–85%) (31). Our data suggest a minor role of *H. influenzae* in severe, spontaneously ruptured otitis media in Germany.

*Streptococcus pyogenes* was the most prevalent bacterial pathogen isolated from MEF throughout the study period, with an *emm*-type distribution similar to that reported for non-invasive disease (32). However, we see a steep reduction in the total cases of *S. pyogenes* AOM, showing a possible impact of PCV use on all-cause AOM, in that, preventing the early *S. pneumoniae* vaccine type episode, there is a reduction in subsequent complex cases caused by non-vaccine serotypes and other otopathogens (21).

*Moraxella catarrhalis* was found in a very low percentage of MEF samples, even though it was prevalent in nasopharyngeal carriage, indicating a minor role of *M. catarrhalis* in spontaneously ruptured otitis media in Germany. The rather high prevalence of *S. aureus* in MEF was probably the result of skin colonization, as testing a subset showed that half of the patients with *S. aureus* in MEF also had *S. aureus* in the healthy ear, whereas this was the case in only 3% of the patients that did not have *S. aureus* isolated from MEF.

Isolates from MEF and NPS were the same in 83 to 99% of all cases, supporting the hypothesis that AOM arises by ascending through the Eustachian tube of the pathogen from the nasopharynx and that carriage precedes AOM (33). This, in turn, underlines the importance of mucosal immunity, mediated by PCVs (34).

Our study shows a high vaccination rate (88%) among children with severe AOM, in accordance with the vaccination rate estimates of the general childhood population in Germany (9). Vaccination with PCV7 and PCV13 was negatively associated with vaccine serotype AOM (5.6 vs. 14.8% and 51.7 vs. 70.4%). Overall, a direct association of *S. pneumoniae* carriage and PCV use could not be shown, but carriage of vaccine type *S. pneumoniae* was lower in vaccinated vs. unvaccinated children. Similar data have been reported from the US (30).

We found it noteworthy that such a high percentage of children had at least one person smoking in the household (16–22%), particularly since these data were self-reported, which could mean that the actual rate of smoking is higher (35, 36). Smoking was associated with AOM from *S. pneumoniae* and remains an important risk factor in pediatric pneumococcal disease. Our study corroborates previous findings that the presence of siblings in the household is common in children with AOM, as well as having siblings in daycare (37–40).

The complex inter- and intraspecies relationships between NPS and MEF organisms described here are of interest: children with any pneumococcal AOM were less likely to be colonized by *S. pyogenes*, while children with AOM caused by *S. pyogenes* were less likely to be colonized by *S. pneumoniae*, which is echoed in the negative association between these two pathogens in nasopharyngeal carriage. The contrast between the apparent niches for *S. pneumoniae* and *S. pyogenes* extends into demographic associations as well: *S. pyogenes* was positively associated with siblings in the household and siblings in daycare; *S. pneumoniae* was negatively associated with both siblings in the household and siblings in daycare. This antipathy is of particular interest given the rise in the proportion of *S. pyogenes* AOM over the course of the study.

## Conclusion

Pneumococcal conjugate vaccination has had a strong impact on the prevalence and etiology of spontaneously ruptured otitis media among children in Germany. Case numbers decreased by over 80%, with *S. pneumoniae* decreasing in prevalence, *S. pyogenes* increasing, and a low prevalence of *M. catarrhalis*. We describe a high degree of correlation between NPS and MEF isolates, showing the strong relationship between colonization and non-invasive disease in these proximate but distinct anatomical sites. As the prevalence of *S. pneumoniae* in carriage shows little change, but vaccine serotypes have virtually disappeared in disease, the replacement serotypes seem to be less capable of causing severe AOM.

## Data Availability Statement

The original contributions presented in the study are included in the article/Supplementary Material, further inquiries can be directed to the corresponding author/s.

## Ethics Statement

The studies involving human participants were reviewed and approved by Ethik-Kommission der Ärztekammer Westfalen-Lippe und der Medizinischen Fakultät der Westfälischen Wilhelms-Universität Münster. Written informed consent to participate in this study was provided by the participants' legal guardian/next of kin.

## Author Contributions

ML, MI, and AB were involved in conception of study. AB was involved in collection of the samples. MI and ML were involved in laboratory analyses of the samples. Data analyses were performed by SP and ML. All authors were involved in writing the manuscript.

## Conflict of Interest

ML and AB have been members of the advisory boards of Pfizer Pharma GmbH Germany. SP had travel fees paid by Pfizer. The remaining author declares that the research was conducted in the absence of any commercial or financial relationships that could be construed as a potential conflict of interest.
