# Supplementary material for: Bacterial Spectrum of Spontaneously Ruptured Otitis Media in a 7-Year, Longitudinal, Multicenter, Epidemiological Cross-Sectional Study in Germany
Source: Front Med (Lausanne). 2021 May 20;8:675225. doi: 10.3389/fmed.2021.675225 (PMC8172772; doi:10.3389/fmed.2021.675225)
Supplement: Supplementary file 1 [file Data_Sheet_1.doc]

**Supplemental Material**

**Supplemental Material** 1: List of participating centers: green: first 43 participating centers (2008-2014), white: additional 32 centers (2010-2014). Map with geographic spread of centers participating in the study. List of recruiting participants.

| **Patients from Centers** | **Center City** | **Y1** | **%** | **Y2** | **%** | **Y3** | **%** | **Y4** | **%** | **Y5** | **%** | **Y6** | **%** | **Y7** | **%** | **total** | **%** |
| --- | --- | --- | --- | --- | --- | --- | --- | --- | --- | --- | --- | --- | --- | --- | --- | --- | --- |
| A29 | Aschaffenburg |  |  |  |  |  |  | 3 | 0.7 |  |  |  |  |  |  | 3 | 0.1 |
| A31 | Asbach |  |  |  |  |  |  | 3 | 0.7 | 1 | 0.3 |  |  |  |  | 4 | 0.2 |
| A32 | Altdorf b. Nürnberg |  |  |  |  |  |  | 7 | 1.7 | 10 | 3.0 | 4 | 1.6 | 11 | 5.3 | 32 | 1.5 |
| A33 | Arnsberg, Westf. |  |  |  |  |  |  | 2 | 0.5 | 1 | 0.3 |  |  |  |  | 3 | 0.1 |
| B100 | Bad Sobernheim | 3 | 0.7 |  |  |  |  |  |  |  |  |  |  |  |  | 3 | 0.1 |
| B101 | Bönnigheim | 5 | 1.1 | 2 | 0.7 | 4 | 1.9 | 2 | 0.5 | 3 | 0.9 | 1 | 0.4 |  |  | 17 | 0.8 |
| B103 | Berlin | 5 | 1.1 | 7 | 2.3 | 3 | 1.4 | 13 | 3.2 | 8 | 2.4 | 9 | 3.7 | 10 | 4.8 | 55 | 2.6 |
| B120 | Bretten |  |  |  |  | 1 | 0.5 | 4 | 1.0 | 1 | 0.3 | 3 | 1.2 | 2 | 1.0 | 11 | 0.5 |
| B121 | Baunatal |  |  |  |  |  |  | 1 | 0.2 |  |  |  |  |  |  | 1 | 0.0 |
| B122 | Berlin |  |  |  |  | 1 | 0.5 | 3 | 0.7 | 2 | 0.6 | 5 | 2.0 |  |  | 11 | 0.5 |
| B123 | Bochum |  |  |  |  | 1 | 0.5 | 11 | 2.7 | 1 | 0.3 |  |  |  |  | 13 | 0.6 |
| B124 | Bocholt |  |  |  |  |  |  | 3 | 0.7 | 3 | 0.9 |  |  | 2 | 1.0 | 8 | 0.4 |
| B125 | Bonn |  |  |  |  |  |  | 1 | 0.2 |  |  |  |  |  |  | 1 | 0.0 |
| B96 | Bramsche | 15 | 3.4 | 7 | 2.3 | 13 | 6.3 | 10 | 2.5 | 5 | 1.5 | 5 | 2.0 | 10 | 4.8 | 65 | 3.0 |
| B97 | Berlin | 22 | 5.0 | 19 | 6.3 |  |  |  |  |  |  |  |  |  |  | 41 | 1.9 |
| C15 | Cham | 10 | 2.3 | 7 | 2.3 | 4 | 1.9 | 6 | 1.5 | 2 | 0.6 |  |  | 3 | 1.4 | 32 | 1.5 |
| D58 | Dortmund |  |  |  |  |  |  | 3 | 0.7 | 1 | 0.3 | 1 | 0.4 | 2 | 1.0 | 7 | 0.3 |
| D59 | Datteln |  |  |  |  | 2 | 1.0 | 15 | 3.7 | 10 | 3.0 | 6 | 2.4 | 6 | 2.9 | 39 | 1.8 |
| E30 | Eschborn | 14 | 3.2 | 9 | 3.0 | 5 | 2.4 | 6 | 1.5 | 4 | 1.2 |  |  |  |  | 38 | 1.8 |
| E33 | Eschwege | 7 | 1.6 | 15 | 5.0 | 14 | 6.7 | 11 | 2.7 | 8 | 2.4 | 15 | 6.1 | 5 | 2.4 | 75 | 3.5 |
| F21 | Freilassing | 6 | 1.4 | 5 | 1.7 | 5 | 2.4 | 5 | 1.2 | 8 | 2.4 | 2 | 0.8 | 2 | 1.0 | 33 | 1.5 |
| F22 | Flensburg | 11 | 2.5 | 3 | 1.0 | 1 | 0.5 | 2 | 0.5 | 3 | 0.9 | 6 | 2.4 | 11 | 5.3 | 37 | 1.7 |
| F25 | Flonheim |  |  |  |  | 2 | 1.0 | 2 | 0.5 |  |  |  |  |  |  | 4 | 0.2 |
| F26 | Frankenthal |  |  |  |  | 1 | 0.5 | 21 | 5.2 | 19 | 5.7 | 25 | 10.2 | 17 | 8.1 | 83 | 3.9 |
| F28 | Frankfurt am Main |  |  |  |  |  |  | 5 | 1.2 | 13 | 3.9 | 2 | 0.8 | 8 | 3.8 | 28 | 1.3 |
| G35 | Grafrath | 9 | 2.1 | 10 | 3.3 | 5 | 2.4 | 2 | 0.5 | 3 | 0.9 | 5 | 2.0 | 1 | 0.5 | 35 | 1.6 |
| G36 | Gau-Odernheim | 6 | 1.4 |  |  |  |  |  |  |  |  |  |  |  |  | 6 | 0.3 |
| G41 | Göttingen |  |  |  |  | 2 | 1.0 | 9 | 2.2 | 3 | 0.9 | 7 | 2.9 | 7 | 3.3 | 28 | 1.3 |
| H63 | Hamm | 9 | 2.1 | 2 | 0.7 | 2 | 1.0 |  |  |  |  |  |  |  |  | 13 | 0.6 |
| H64 | Herzogenaurach | 5 | 1.1 | 3 | 1.0 |  |  |  |  |  |  |  |  |  |  | 8 | 0.4 |
| H65 | Hamburg | 11 | 2.5 | 10 | 3.3 | 2 | 1.0 | 6 | 1.5 | 1 | 0.3 |  |  |  |  | 30 | 1.4 |
| H66 | Heilbronn | 13 | 3.0 | 11 | 3.7 | 7 | 3.4 | 2 | 0.5 | 2 | 0.6 | 4 | 1.6 | 1 | 0.5 | 40 | 1.9 |
| K47 | Kehl | 5 | 1.1 |  |  |  |  |  | 0.0 |  |  |  |  |  |  | 5 | 0.2 |
| K48 | Kempten | 9 | 2.1 | 4 | 1.3 | 2 | 1.0 | 1 | 0.2 | 4 | 1.2 | 10 | 4.1 | 2 | 1.0 | 32 | 1.5 |
| K49 | Krefeld | 8 | 1.8 | 7 | 2.3 | 4 | 1.9 |  |  |  |  |  |  |  |  | 19 | 0.9 |
| K50 | Kleve | 1 | 0.2 | 4 | 1.3 |  |  |  |  |  |  |  |  |  |  | 5 | 0.2 |
| K58 | Kassel |  |  |  |  |  |  | 4 | 1.0 |  |  |  |  |  |  | 4 | 0.2 |
| K59 | Kaufbeuren |  |  |  |  |  |  | 2 | 0.5 | 2 | 0.6 | 2 | 0.8 |  |  | 6 | 0.3 |
| K60 | Kassel |  |  |  |  |  |  | 15 | 3.7 | 15 | 4.5 | 8 | 3.3 | 2 | 1.0 | 40 | 1.9 |
| L38 | Lübeck | 14 | 3.2 | 13 | 4.3 | 7 | 3.4 | 12 | 3.0 | 8 | 2.4 | 12 | 4.9 | 13 | 6.2 | 79 | 3.7 |
| L50 | Lauffen am Neckar |  |  |  |  |  |  | 1 | 0.2 |  |  |  |  |  |  | 1 | 0.0 |
| M55 | München | 4 | 0.9 |  |  |  |  | 8 | 2.0 | 10 | 3.0 | 1 | 0.4 |  |  | 23 | 1.1 |
| M56 | Minden | 8 | 1.8 | 6 | 2.0 |  |  |  |  |  |  |  |  |  |  | 14 | 0.7 |
| M59 | Münster |  |  |  |  | 1 | 0.5 | 5 | 1.2 | 3 | 0.9 | 2 | 0.8 |  |  | 11 | 0.5 |
| M60 | München |  |  |  |  |  |  | 2 | 0.5 | 4 | 1.2 | 3 | 1.2 | 1 | 0.5 | 10 | 0.5 |
| M62 | Mainz |  |  |  |  |  |  | 7 | 1.7 | 6 | 1.8 | 1 | 0.4 | 3 | 1.4 | 17 | 0.8 |
| M63 | Münster |  |  |  |  |  |  | 2 | 0.5 | 5 | 1.5 |  |  |  |  | 7 | 0.3 |
| N19 | Neumünster | 5 | 1.1 | 9 | 3.0 | 6 | 2.9 | 10 | 2.5 | 2 | 0.6 | 1 | 0.4 |  |  | 33 | 1.5 |
| N21 | Neustadt/Aisch | 20 | 4.6 | 13 | 4.3 | 12 | 5.8 | 14 | 3.5 | 14 | 4.2 | 9 | 3.7 | 2 | 1.0 | 84 | 3.9 |
| N22 | Neumünster | 12 | 2.7 | 4 | 1.3 | 1 | 0.5 | 3 | 0.7 | 4 | 1.2 | 2 | 0.8 | 2 | 1.0 | 28 | 1.3 |
| N23 | Neumünster | 6 | 1.4 | 4 | 1.3 | 2 | 1.0 |  |  |  |  |  |  |  |  | 12 | 0.6 |
| N24 | Neumünster | 9 | 2.1 | 7 | 2.3 | 7 | 3.4 | 1 | 0.2 | 7 | 2.1 | 4 | 1.6 | 10 | 4.8 | 45 | 2.1 |
| N27 | Nürnberg |  |  |  |  |  |  | 30 | 7.4 | 29 | 8.7 | 16 | 6.5 | 12 | 5.7 | 87 | 4.1 |
| O14 | Oberkirch | 10 | 2.3 | 18 | 6.0 | 4 | 1.9 | 13 | 3.2 | 22 | 6.6 | 11 | 4.5 | 10 | 4.8 | 88 | 4.1 |
| O15 | Olching | 1 | 0.2 |  |  | 8 | 3.8 | 2 | 0.5 | 3 | 0.9 | 3 | 1.2 |  |  | 17 | 0.8 |
| O18 | Offenbach |  |  |  |  |  |  | 16 | 4.0 | 5 | 1.5 | 4 | 1.6 | 1 | 0.5 | 26 | 1.2 |
| O20 | Oberstenfeld |  |  |  |  |  |  | 8 | 2.0 | 8 | 2.4 | 2 | 0.8 |  |  | 18 | 0.8 |
| P19 | Prien | 14 | 3.2 | 20 | 6.7 | 24 | 11.5 | 11 | 2.7 | 13 | 3.9 | 12 | 4.9 | 5 | 2.4 | 99 | 4.6 |
| P20 | Porta Westfalica | 15 | 3.4 | 7 | 2.3 | 3 | 1.4 |  |  |  |  | 1 | 0.4 | 1 | 0.5 | 27 | 1.3 |
| R23 | Rosenheim | 7 | 1.6 | 4 | 1.3 | 1 | 0.5 |  |  | 1 | 0.3 | 1 | 0.4 | 4 | 1.9 | 18 | 0.8 |
| S42 | Schwieberdingen | 10 | 2.3 | 6 | 2.0 | 6 | 2.9 | 5 | 1.2 | 4 | 1.2 | 5 | 2.0 | 1 | 0.5 | 37 | 1.7 |
| S46 | Solingen |  |  |  |  | 1 | 0.5 | 6 | 1.5 | 4 | 1.2 | 1 | 0.4 | 2 | 1.0 | 14 | 0.7 |
| S51 | Saulgau | 2 | 0.5 |  |  |  |  |  |  |  |  |  |  |  |  | 2 | 0.1 |
| S52 | Schwäbisch-Hall | 38 | 8.7 | 20 | 6.7 | 2 | 1.0 | 3 | 0.7 | 7 | 2.1 |  |  | 3 | 1.4 | 73 | 3.4 |
| T10 | Traunreut | 18 | 4.1 | 7 | 2.3 | 14 | 6.7 | 4 | 1.0 | 10 | 3.0 | 7 | 2.9 | 7 | 3.3 | 67 | 3.1 |
| T2 | Tegernsee | 3 | 0.7 | 1 | 0.3 | 2 | 1.0 | 1 | 0.2 |  |  |  |  |  |  | 7 | 0.3 |
| V8 | Vellmar |  |  |  |  |  |  | 3 | 0.7 | 4 | 1.2 | 1 | 0.4 | 1 | 0.5 | 9 | 0.4 |
| W34 | Würzburg | 16 | 3.6 | 13 | 4.3 | 6 | 2.9 | 2 | 0.5 |  |  |  |  |  |  | 37 | 1.7 |
| W35 | Weiden i.d.Opf. | 9 | 2.1 | 2 | 0.7 | 6 | 2.9 | 6 | 1.5 |  |  | 3 | 1.2 | 7 | 3.3 | 33 | 1.5 |
| W36 | Wiesbaden | 17 | 3.9 |  |  |  |  | 1 | 0.2 | 4 | 1.2 |  |  |  |  | 22 | 1.0 |
| W37 | Welzheim | 21 | 4.8 | 12 | 4.0 | 10 | 4.8 | 11 | 2.7 | 9 | 2.7 | 4 | 1.6 | 8 | 3.8 | 75 | 3.5 |
| W38 | Weilheim | 6 | 1.4 | 9 | 3.0 | 1 | 0.5 | 1 | 0.2 | 2 | 0.6 |  |  |  |  | 19 | 0.9 |
| W49 | Wilhelmshaven |  |  |  |  |  |  | 11 | 2.7 | 4 | 1.2 | 3 | 1.2 | 2 | 1.0 | 20 | 0.9 |
| W50 | Würselen |  |  |  |  | 3 | 1.4 | 14 | 3.5 | 3 | 0.9 | 11 | 4.5 | 9 | 4.3 | 40 | 1.9 |
| W51 | Würzburg |  |  |  |  |  |  | 11 | 2.7 | 5 | 1.5 | 5 | 2.0 | 3 | 1.4 | 24 | 1.1 |
| Total |  | 439 | 100.0 | 300 | 100.0 | 208 | 100.0 | 404 | 100.0 | 333 | 100.0 | 245 | 100.0 | 209 | 100.0 | 2138 | 100.0 |
| Total original centers |  | 439 | 100.0 | 300 | 100.0 | 193 | 92.8 | 174 | 43.1 | 171 | 51.4 | 133 | 54.3 | 118 | 56.5 | 1528 | 71.5 |
| Total additional centers |  | 0 | 0.0 | 0 | 0.0 | 15 | 7.2 | 230 | 56.9 | 162 | 48.6 | 112 | 45.7 | 91 | 43.5 | 610 | 28.5 |

**We would like to thank the following pediatricians for their excellent collaboration in collecting samples.**

| **Title** | **First name** | **Last name** | **City** |
| --- | --- | --- | --- |
| Dr. med. | Dominik | **Fürsich** | Altdorf b. Nürnberg |
| Dr. med. | Burkhard | **Lawrenz** | Arnsberg |
| Dr. med. | Andreas | **Zimmermann** | Asbach |
| Dr. med. | Bernhard | **Sandner** | Aschaffenburg |
| Dr. med. | Rainer | **Lauf** | Bad Sobernheim |
| Dr. med. | Gerhard | **Bleckmann** | Baunatal |
| Dr. med. | Klaus-Jürgen | **Taube** | Berlin |
| Dr. med. | Susanne | **Blume** | Berlin |
| Dr. med. | Petra | **van Stiphout** | Berlin |
| Dr. med. | Heinrich | **Nolte** | Bocholt |
| Dr. med. | Thomas | **Beck** | Bochum |
| Prof. Dr. med. | Alireza | **Ranjbar** | Bonn |
| Dr. med. | Renate | **Mangeldorf-Taxis** | Bönnigheim |
| Dr. med. | Thomas | **Adelt** | Bramsche |
| Dr. med. | Roland | **Knecht** | Bretten |
| Dr. med. | Thomas | **Habash** | Cham |
| Dr. med. | Andreas | **Schmutte** | Datteln |
| Dr. med. | Harald | **Neugebauer** | Dortmund |
| Dr. med. | Geri | **Lichtenstein** | Eschborn |
| Dr. med. | Florian | **Peters** | Eschwege |
| Dr. med. | Geert | **Morf** | Flensburg |
| Dr. med. | Hans-Joachim | **Büttner** | Flonheim, Gau-Odernheim |
| Dr. med. | Lothar | **Maurer** | Frankenthal |
| Dr. med. | Burkhard | **Voigt** | Frankfurt am Main |
| Dr. med. | Jürgen | **Geuder** | Freilassing |
|  | Daniela | **Kober** | Gmund |
| Dr. med. | Mathias | **Feindt** | Göttingen |
| Dr. med. | Susanne | **Linder** | Grafrath |
| Dr. med. | Hans-Christoph | **Runge** | Hamburg |
| Dr. med. | Hans-Ulrich | **Henschel** | Hamm |
| Dr. med. | Hans Ulrich | **Stechele** | Heilbronn |
|  | Peter | **Lautenbach** | Herzogenaurach |
| Dr. med. | Lars | **Nelleßen** | Kassel |
|  | Alfons | **Fleer** | Kassel |
| Dr. med. | Volkmar | **Reschke** | Kaufbeuren |
| Dr. med. | Ulrich | **Behre** | Kehl |
| Dr. med. | Dietrich | **Grunert** | Kempten |
| Dr. med. | Peter | **Soemantri** | Kleve |
| Dr. med. | Karl-Heinz | **Laakmann** | Krefeld |
| Dr. med. | Michael | **Mühlschlegel** | Lauffen am Neckar |
| Dr. med. | Sobhi | **Mahdi** | Lübeck |
| Dr. med. | Pirmin | **Habermehl** | Mainz |
| Dr. med. | Helmut | **Wolschner** | Minden |
| Dr. med. | Matthias | **Grimberg** | München |
|  | Philipp | **Schoof** | München |
| Dr. med. | Janbernd | **Neuhann** | Münster |
|  | Burkhard | **Frase** | Münster |
|  | Rolf | **Clementsen** | Neumünster |
| Dr. med. | Petra | **Mahlo** | Neumünster |
| Dipl. med. | Sabine | **Maruschke** | Neumünster |
| Dr. med. | Johannes | **Kandzora** | Neumünster |
| Dr. med. | Dietrich | **Distel** | Neustadt / Aisch |
| Dr. med. | Wolfgang | **Landendörfer** | Nürnberg |
| Dr. med. | Hanns-Michael | **Burow** | Oberkirch |
| Dr. med. | Martin | **Kimmig** | Oberstenfeld |
| Dr. med. | Matthias | **Gründler** | Offenbach am Main |
| Dr. med. | Christian | **Pauli** | Olching |
| Dr. med. | Stefan | **Noll** | Porta Westfalica |
| Dr. med. | Heinz | **Lechner** | Prien |
|  | Otto | **Laub** | Rosenheim |
| Dr. med. | Michael | **Steiner** | Saulgau |
| Dr. med. | Barbara | **Möhlig** | Schwäbisch Hall |
| Dr. med. | Sylvia | **Scherl** | Schwieberdingen |
| Dr. med. | Thomas | **Fischbach** | Solingen |
| Dr. med. | Andreas | **Busse** | Tegernsee |
| Dr. med. | Karl | **Landvogt** | Traunreut |
|  | Thomas | **Lenz** | Vellmar |
| Dr. med. | Egbert | **Leonhardt** | Weiden i.d. Opf. |
| Dr. med. | Christoph | **Wittermann** | Weilheim |
| Dr. med. | Dieter | **Schlegel** | Welzheim |
| Dr. med. | Elmar | **Schäfer** | Wiesbaden |
| Dr. med. | Rupert | **Dernick** | Wilhelmshaven |
| Dr. med. | Claus | **Balz-Herrmann** | Würselen |
| Dr. med. | Wolfgang | **Brosi** | Würzburg |
| Dr. med. | Roland | **Schleupner** | Würzburg |

**Supplemental material 2:** List of pathogens found in MEF isolates. Yellow: Relevant AOM pathogens analysed in this study, white: other pathogens not analysed in this study, green: normal ear flora, red: no growth.

| **Species** | **Y1** | **%** | **Y2** | **%** | **Y3** | **%** | **Y4** | **%** | **Y5** | **%** | **Y6** | **%** | **Y7** | **%** | **All** | **%** |
| --- | --- | --- | --- | --- | --- | --- | --- | --- | --- | --- | --- | --- | --- | --- | --- | --- |
| Haemophilus influenzae | 31 | 7.1 | 17 | 5.7 | 14 | 6.7 | 26 | 6.4 | 15 | 4.5 | 18 | 7.3 | 12 | 5.7 | 133 | 6.2 |
| Moraxella catarrhalis | 7 | 1.6 | 1 | 0.3 |  |  | 1 | 0.2 |  |  | 1 | 0.4 |  |  | 10 | 0.5 |
| Staphylococcus aureus | 36 | 8.2 | 23 | 7.7 | 15 | 7.2 | 33 | 8.2 | 23 | 6.9 | 20 | 8.2 | 18 | 8.6 | 168 | 7.9 |
| Streptococcus pneumoniae | 43 | 9.8 | 35 | 11.7 | 12 | 5.8 | 28 | 6.9 | 21 | 6.3 | 17 | 6.9 | 14 | 6.7 | 170 | 8.0 |
| Streptococcus pyogenes | 53 | 12.1 | 34 | 11.3 | 24 | 11.5 | 75 | 18.6 | 60 | 18.0 | 36 | 14.7 | 33 | 15.8 | 315 | 14.7 |
| **Total relevant isolates** | **170** | **38.7** | **110** | **36.7** | **65** | **31.3** | **163** | **40.3** | **119** | **35.7** | **92** | **37.6** | **77** | **36.8** | **796** | **37.2** |
| Escherichia coli | 2 | 0.5 | 1 | 0.3 | 2 | 1.0 | 1 | 0.2 |  |  | 1 | 0.4 | 1 | 0.5 | 8 | 0.4 |
| Klebsiella pneumoniae | 3 | 0.7 |  |  |  |  |  |  |  |  |  |  |  |  | 3 | 0.1 |
| Proteus mirabilis |  |  |  |  |  |  | 1 | 0.2 |  |  |  |  | 2 | 1.0 | 3 | 0.1 |
| Pseudomonas aeruginosa | 12 | 2.7 | 9 | 3.0 | 3 | 1.4 | 9 | 2.2 | 3 | 0.9 | 2 | 0.8 | 1 | 0.5 | 39 | 1.8 |
| Serratia marcescens | 1 | 0.2 | 1 | 0.3 |  |  |  |  |  |  |  |  |  |  | 2 | 0.1 |
| **Total other isolates with known pathogenic potential** | **18** | **4.1** | **11** | **3.7** | **5** | **2.4** | **11** | **2.7** | **3** | **0.9** | **3** | **1.2** | **4** | **1.9** | **55** | **2.6** |
| Candida lusitaniae |  |  |  |  |  |  | 1 | 0.2 |  |  |  |  |  |  | 1 | 0.05 |
| Candida parapsilosis |  |  |  |  | 1 | 0.5 |  |  |  |  |  |  |  |  | 1 | 0.05 |
| Enterococcus faecalis | 6 | 1.4 | 4 | 1.3 |  |  | 1 | 0.2 |  |  |  |  | 3 | 1.4 | 14 | 0.7 |
| Enterococcus faecium | 2 | 0.5 | 1 | 0.3 |  |  |  |  |  |  |  |  |  |  | 3 | 0.1 |
| Turicella otitidis |  |  |  |  |  |  | 2 | 0.5 | 1 | 0.3 | 1 | 0.4 | 3 | 1.4 | 7 | 0.3 |
| **Total questionable pathogenic potential** | **8** | **1.8** | **5** | **1.7** | **1** | **0.5** | **4** | **1.0** | **1** | **0.3** | **1** | **0.4** | **6** | **2.9** | **26** | **1.2** |
| Achromobacter xylosidans |  |  |  |  |  |  |  |  |  |  | 1 | 0.4 |  |  | 1 | 0.05 |
| Acinetobacter baumannii | 3 | 0.7 |  |  |  |  |  |  |  |  |  |  |  |  | 3 | 0.1 |
| Acinetobacter baumanii complex |  |  |  |  |  |  |  |  |  |  |  |  | 1 | 0.5 | 1 | 0.05 |
| Acinetobacter genomospecies 3 |  |  |  |  |  |  | 3 | 0.7 |  |  |  |  |  |  | 3 | 0.1 |
| Acinetobacter johnsonii |  |  |  |  |  |  | 1 | 0.2 |  |  |  |  | 2 | 1.0 | 3 | 0.1 |
| Acinetobacter lwoffii |  |  | 2 | 0.7 | 2 | 1.0 | 4 | 1.0 |  |  | 1 | 0.4 | 1 | 0.5 | 10 | 0.5 |
| Acinetobacter radioresistens |  |  |  |  |  |  |  |  |  |  |  |  | 2 | 1.0 | 2 | 0.1 |
| Acinetobacter sp. |  |  |  |  | 2 | 1.0 |  |  |  |  |  |  |  |  | 2 | 0.1 |
| Alcaligenes faecalis | 1 | 0.2 |  |  |  |  |  |  |  |  |  |  |  |  | 1 | 0.05 |
| Arthrobacter gandavensis |  |  |  |  |  |  | 1 | 0.2 |  |  |  |  |  |  | 1 | 0.05 |
| Bacillus cereus | 9 | 2.1 | 13 | 4.3 | 2 | 1.0 | 5 | 1.2 | 3 | 0.9 |  |  | 3 | 1.4 | 35 | 1.6 |
| Bacillus licheniformis |  |  |  |  |  |  | 1 | 0.2 |  |  | 1 | 0.4 |  |  | 2 | 0.1 |
| Bacillus pumilus |  |  |  |  | 3 | 1.4 | 3 | 0.7 |  |  |  |  | 1 | 0.5 | 7 | 0.3 |
| Bacillus simplex |  |  |  |  |  |  | 1 | 0.2 |  |  |  |  |  |  | 1 | 0.05 |
| Bacillus sp. | 5 | 1.1 | 6 | 2.0 | 4 | 1.9 | 5 | 1.2 | 3 | 0.9 |  |  | 1 | 0.5 | 24 | 1.1 |
| Bacillus subtilis |  |  |  |  |  |  | 1 | 0.2 |  |  |  |  | 1 | 0.5 | 2 | 0.1 |
| Bacillus thuringiensis |  |  |  |  | 1 | 0.5 | 1 | 0.2 |  |  |  |  | 1 | 0.5 | 3 | 0.1 |
| Bacillus weihenstephanenesis |  |  |  |  |  |  |  |  |  |  |  |  | 1 | 0.5 | 1 | 0.05 |
| Citrobacter braakii |  |  |  |  |  |  | 1 | 0.2 |  |  |  |  |  |  | 1 | 0.05 |
| Citrobacter freundii |  |  | 1 | 0.3 |  |  | 1 | 0.2 |  |  |  |  |  |  | 2 | 0.1 |
| Comamonas testosteroni | 1 | 0.2 |  |  |  |  |  |  |  |  |  |  |  |  | 1 | 0.05 |
| Enterobacter cloacae | 2 | 0.5 | 2 | 0.7 | 3 | 1.4 | 1 | 0.2 |  |  |  |  |  |  | 8 | 0.4 |
| Enterobacter kobei |  |  |  |  |  |  | 1 | 0.2 |  |  |  |  |  |  | 1 | 0.05 |
| Enterobacter sp. |  |  |  |  |  |  |  |  | 1 | 0.3 |  |  |  |  | 1 | 0.05 |
| Enterobacter cloacae complex |  |  |  |  |  |  |  |  |  |  | 1 | 0.4 | 1 | 0.5 | 2 | 0.1 |
| Lactobacillus sakei |  |  | 1 | 0.3 |  |  |  |  |  |  |  |  |  |  | 1 | 0.05 |
| Klebsiella oxytoca | 3 | 0.7 |  |  |  |  | 2 | 0.5 |  |  |  |  |  |  | 5 | 0.2 |
| Micrococcus liquefaciens |  |  |  |  |  |  |  |  |  |  |  |  | 1 | 0.5 | 1 | 0.05 |
| Micrococcus luteus |  |  |  |  |  |  |  |  |  |  |  |  | 2 | 1.0 | 2 | 0.1 |
| Moraxella osloensis |  |  |  |  |  |  | 1 | 0.2 |  |  |  |  |  |  | 1 | 0.05 |
| Moraxella sp. |  |  |  |  |  |  |  | 0.0 | 1 | 0.3 |  |  |  |  | 1 | 0.05 |
| Pantoea agglomerans | 2 | 0.5 | 2 | 0.7 | 1 | 0.5 | 1 | 0.2 |  |  |  |  |  |  | 6 | 0.3 |
| Pseudomonas oryzihabitans | 1 | 0.2 |  |  |  |  |  |  |  |  |  |  |  |  | 1 | 0.05 |
| Pseudomonas stutzeri | 1 | 0.2 | 2 | 0.7 | 2 | 1.0 |  |  | 1 | 0.3 |  |  |  |  | 6 | 0.3 |
| Stenotrophomonas maltophilia |  |  |  |  |  |  | 2 | 0.5 |  |  | 1 | 0.4 |  |  | 3 | 0.1 |
| **Total temporary contamination of auditory canal** | **28** | **1.3** | **29** | **1.4** | **20** | **0.9** | **36** | **1.7** | **9** | **0.4** | **5** | **0.2** | **18** | **0.8** | **145** | **6.8** |
| Propionibacterium sp. | 1 | 0.2 |  |  |  |  |  |  |  |  |  |  |  |  | 1 | 0.05 |
| Corynebacterium amycolatum | 1 | 0.2 |  |  |  |  |  |  |  |  |  |  |  |  | 1 | 0.05 |
| Corynebacterium argentoratense |  |  |  |  |  |  |  |  |  |  |  |  | 1 | 0.5 | 1 | 0.05 |
| Corynebacterium sp. | 2 | 0.5 | 2 | 0.7 |  |  |  |  |  |  |  |  |  |  | 4 | 0.2 |
| coagulase negative Staphylococci | 105 | 23.9 | 88 | 29.3 | 82 | 39.4 | 149 | 36.9 | 167 | 50.2 | 98 | 40.0 | 31 | 14.8 | 720 | 33.7 |
| viridans Streptococci | 7 | 1.6 | 11 | 3.7 | 4 | 1.9 | 7 | 1.7 | 5 | 1.5 | 2 | 0.8 |  |  | 36 | 1.7 |
| other normal ear flora | 43 | 9.8 |  |  |  |  |  |  |  |  | 5 | 2.0 | 54 | 25.8 | 151 | 7.1 |
| **Total normal ear flora** | **159** | **36.2** | **150** | **34.2** | **86** | **19.6** | **156** | **35.5** | **172** | **39.2** | **105** | **23.9** | **86** | **19.6** | **914** | **208.2** |
| **no relevant bacterial growth** | **62** | **14.1** | **49** | **16.3** | **32** | **15.4** | **42** | **10.4** | **36** | **10.8** | **45** | **18.4** | **23** | **11.0** | **240** | **11.2** |
| **Total isolates** | **445** | **101.4** | **305** | **101.7** | **209** | **100.5** | **412** | **102.0** | **340** | **102.1** | **251** | **102.4** | **214** | **102.4** | **2176** | **101.8** |
| **Patients with two isolates** | **6** | **1.4** | **5** | **1.7** | **1** | **0.5** | **8** | **2.0** | **7** | **2.1** | **6** | **2.4** | **5** | **2.4** | **38** | **1.8** |
| **Total patients** | **439** | **100.0** | **300** | **100.0** | **208** | **100.0** | **404** | **100.0** | **333** | **100.0** | **245** | **100.0** | **209** | **100.0** | **2138** | **100.0** |

**Supplemental material 3:** Comparison of pathogens found in MEF and NPS of the same patient. Green: Same serotype found in MEF and NPS.

| ***S. pneumoniae* serotype** | **n=** | **%** | **% of NPS Spn positive** |
| --- | --- | --- | --- |
| MEF=NPS | 114 | 67.5 | 91.9 |
| MEF≠NPS | 10 | 5.9 | 8.1 |
| no serotype in MEF | 4 | 2.4 |  |
| no *S. pneumoniae* in NPS | 41 | 24.3 |  |
| MEF=non-infected ear | 1 | 0.6 |  |
| **Total** | **169*** | **100.0** |  |

* One patient had two *S. pneumoniae* in MEF

| **Patient** | **MEF** | | **NPS** | | **comment** |
| --- | --- | --- | --- | --- | --- |
|  | **1^st^ serotype** | **2^nd^ serotype** | **1^st^ serotype** | **2^nd^ serotype** |  |
| 1119 | 1 |  | 1 |  |  |
| 0106 | 3 |  | 3 |  |  |
| 0112 | 3 |  | 3 |  |  |
| 0159 | 3 |  | 3 |  |  |
| 0627 | 3 |  | 3 |  |  |
| 1165 | 3 |  | 3 |  |  |
| 1167 | 3 |  | 3 | 11A | 2 *S. pneumoniae* in NPS |
| 1210 | 3 |  | 3 |  |  |
| 1411 | 3 |  | 3 |  |  |
| 1412 | 3 |  | 3 |  |  |
| 1519 | 3 |  | 3 |  |  |
| 1723 | 3 |  | 3 |  |  |
| 2311 | 3 |  | 3 | 23A | 2 *S. pneumoniae* in NPS |
| 2322 | 3 |  | 3 |  |  |
| 2324 | 3 |  | 3 |  |  |
| 2501 | 3 | 23B | 3 |  | 2 *S. pneumoniae* in MEF |
| 2534 | 3 |  | 3 | 11A | 2 *S. pneumoniae* in NPS |
| 2604 | 3 |  | 3 |  |  |
| 2615 | 3 |  | 3 |  |  |
| 3019 | 3 |  | 3 |  |  |
| 3145 | 3 |  | 3 |  |  |
| 3212 | 3 |  | 3 |  |  |
| 3609 | 3 |  | 3 |  |  |
| 3640 | 3 |  | 3 |  |  |
| 3663 | 3 |  | 3 | 35B | 2 *S. pneumoniae* in NPS |
| 4012 | 3 |  | 3 |  |  |
| 4105 | 3 |  | 3 |  |  |
| 4201 | 3 |  | 3 |  |  |
| 4236 | 3 |  | 3 |  |  |
| 4404 | 3 |  | 3 |  |  |
| 4446 | 3 |  | 3 |  |  |
| 4737 | 3 |  | 3 |  |  |
| 5810 | 3 |  | 3 |  |  |
| 5906 | 3 |  | 3 |  |  |
| 6303 | 3 |  | 3 | 11A | 2 *S. pneumoniae* in NPS |
| 6702 | 3 |  | 3 | 22F | 2 *S. pneumoniae* in NPS |
| 6727 | 3 |  | 3 |  |  |
| 6918 | 3 |  | 3 |  |  |
| 6957 | 3 |  | 3 | 10A | 2 *S. pneumoniae* in NPS |
| 6982 | 3 |  | 3 |  |  |
| 7013 | 3 |  | 3 |  |  |
| 7111 | 3 |  | 3 |  |  |
| 8321 | 3 |  | 3 |  |  |
| 0416 | 21 |  | 21 |  |  |
| 1510 | 21 |  | 21 |  |  |
| 4073 | 21 |  | 21 |  |  |
| 0702 | 31 |  | 31 |  |  |
| 7010 | 38 |  | 38 |  |  |
| 5910 | 10A |  | 10A |  |  |
| 6988 | 10A |  | 10A |  |  |
| 0423 | 11A |  | 11A |  |  |
| 1160 | 11A |  | 11A |  |  |
| 1416 | 11A |  | 11A |  |  |
| 1619 | 11A |  | 11A |  |  |
| 4751 | 11A |  | 11A |  |  |
| 5916 | 11A |  | 11A |  |  |
| 6104 | 11A |  | 11A |  |  |
| 6507 | 11A |  | 11A |  |  |
| 7301 | 11A |  | 11A |  |  |
| 5211 | 12F |  | 12F | 21 | 2 *S. pneumoniae* in NPS |
| 0328 | 15B |  | 15B |  |  |
| 0641 | 15C |  | 15C |  |  |
| 2328 | 15C |  | 15C |  |  |
| 3658 | 15C |  | 15C |  |  |
| 5808 | 15C |  | 15C |  |  |
| 0125 | 19A |  | 19A |  |  |
| 0139 | 19A |  | 19A |  |  |
| 0304 | 19A |  | 19A |  |  |
| 0326 | 19A |  | 19A |  |  |
| 0810 | 19A |  | 19A |  |  |
| 0811 | 19A |  | 19A |  |  |
| 1104 | 19A |  | 19A |  |  |
| 1110 | 19A |  | 19A |  |  |
| 1144 | 19A |  | 19A |  |  |
| 2329 | 19A |  | 19A |  |  |
| 2343 | 19A |  | 19A |  |  |
| 3134 | 19A |  | 19A |  |  |
| 3415 | 19A |  | 19A |  |  |
| 3801 | 19A |  | 19A |  |  |
| 3814 | 19A |  | 19A |  |  |
| 3901 | 19A |  | 19A |  |  |
| 4720 | 19A |  | 19A |  |  |
| 6701 | 19A |  | 19A |  |  |
| 6707 | 19A |  | 19A |  |  |
| 6910 | 19A |  | 19A |  |  |
| 6922 | 19A |  | 19A |  |  |
| 0604 | 19F |  | 19F |  |  |
| 0677 | 19F |  | 19F |  |  |
| 0707 | 19F |  | 19F |  |  |
| 2393 | 19F |  | 19F |  |  |
| 2520 | 19F |  | 19F |  |  |
| 6201 | 19F |  | 19F |  |  |
| 7009 | 19F |  | 19F |  |  |
| 4103 | 22F |  | 22F |  |  |
| 6806 | 22F |  | 22F |  |  |
| 0301 | 23A |  | 23A |  |  |
| 2909 | 23A |  | 23A |  |  |
| 0152 | 23B |  | 23B |  |  |
| 2202 | 23B |  | 23B |  |  |
| 3815 | 23B |  | 23B |  |  |
| 2632 | 23F |  | 23F |  |  |
| 5807 | 28A |  | 28A |  |  |
| 1408 | 33F |  | 33F |  |  |
| 4056 | 33F |  | 33F |  |  |
| 7709 | 33F |  | 33F |  |  |
| 3816 | 35B |  | 35B |  |  |
| 4457 | 35B |  | 35B |  |  |
| 5109 | 35B |  | 35B |  |  |
| 2879 | 35C |  | 35C |  |  |
| 4006 | 35F |  | 35F |  |  |
| 6943 | 35F |  | 35F |  |  |
| 0816 | 6C |  | 6C |  |  |
| 4410 | 6C |  | 6C |  |  |
| 0813 | 7F |  | 7F |  |  |
| 0332 | 1 |  | 6A |  |  |
| 2538 | 3 |  | 10A |  |  |
| 4102 | 3 |  | 33F |  |  |
| 3832 | 27 |  | 15C |  |  |
| 5362 | 15A |  | 21 |  |  |
| 6603 | 15C |  | 15B |  |  |
| 6973 | 15C |  | 15B |  |  |
| 1128 | 19F |  | 19A |  |  |
| 6414 | 35B |  | 6C |  |  |
| 4911 | 7F |  | 23F |  |  |
| 1506 |  |  | 34 |  | *S. pneumoniae* in MEF not serotyped |
| 6930 |  |  | 3 |  | *S. pneumoniae* in MEF not serotyped |
| 1205 |  |  | 3 |  | *S. pneumoniae* in MEF not serotyped |
| 2428 |  |  |  |  | *S. pneumoniae* in MEF not serotyped, no NPS obtained |
| 3808 | 1 |  |  |  | no *S. pneumoniae* in NPS |
| 4215 | 1 |  |  |  | no *S. pneumoniae* in NPS |
| 7811 | 1 |  |  |  | no *S. pneumoniae* in NPS |
| 0431 | 3 |  |  |  | no *S. pneumoniae* in NPS |
| 1180 | 3 |  |  |  | no *S. pneumoniae* in NPS |
| 1231 | 3 |  |  |  | no *S. pneumoniae* in NPS |
| 1233 | 3 |  |  |  | no *S. pneumoniae* in NPS |
| 1728 | 3 |  |  |  | no *S. pneumoniae* in NPS |
| 1733 | 3 |  |  |  | no *S. pneumoniae* in NPS |
| 2005 | 3 |  |  |  | no *S. pneumoniae* in NPS |
| 2413 | 3 |  |  |  | no NPS obtained |
| 2839 | 3 |  |  |  | no NPS obtained, *S. pneumoniae* serotype 3 in non-infected ear |
| 3501 | 3 |  |  |  | no NPS obtained |
| 3503 | 3 |  |  |  | no NPS obtained |
| 3660 | 3 |  |  |  | no NPS obtained |
| 6003 | 3 |  |  |  | no NPS obtained |
| 3407 | 21 |  |  |  | no *S. pneumoniae* in NPS |
| 3235 | 31 |  |  |  | no *S. pneumoniae* in NPS |
| 2405 | 11A |  |  |  | no *S. pneumoniae* in NPS |
| 5361 | 11A |  |  |  | no NPS obtained |
| 1103 | 15A |  |  |  | no *S. pneumoniae* in NPS |
| 4445 | 15B |  |  |  | no *S. pneumoniae* in NPS |
| 6125 | 16F |  |  |  | no *S. pneumoniae* in NPS |
| 0401 | 19A |  |  |  | no *S. pneumoniae* in NPS |
| 0414 | 19A |  |  |  | no NPS obtained |
| 0419 | 19A |  |  |  | no NPS obtained |
| 2010 | 19A |  |  |  | no NPS obtained |
| 3506 | 19A |  |  |  | no NPS obtained |
| 3511 | 19A |  |  |  | no NPS obtained |
| 5805 | 19A |  |  |  | no *S. pneumoniae* in NPS |
| 6120 | 19A |  |  |  | no *S. pneumoniae* in NPS |
| 0607 | 19F |  |  |  | no *S. pneumoniae* in NPS |
| 2408 | 19F |  |  |  | no NPS obtained |
| 2801 | 19F |  |  |  | no *S. pneumoniae* in NPS |
| 4752 | 19F |  |  |  | no *S. pneumoniae* in NPS |
| 3657 | 23A |  |  |  | no *S. pneumoniae* in NPS |
| 4403 | 23A |  |  |  | no *S. pneumoniae* in NPS |
| 3215 | 23B |  |  |  | no *S. pneumoniae* in NPS |
| 5377 | 23B |  |  |  | no *S. pneumoniae* in NPS |
| 3637 | 7F |  |  |  | no *S. pneumoniae* in NPS |
| 4412 | NT |  |  |  | no *S. pneumoniae* in NPS |

| ***H. influenzae* serotype** | **n=** | **%** | **% of NPS positive** |
| --- | --- | --- | --- |
| MEF=NPS | 80 | 60.2 | 83.3 |
| MEF≠NPS | 16 | 12.0 | 16.7 |
| no serotype in MEF | 4 | 3.0 |  |
| no *H. Influenzae* in NPS | 33 | 24.8 |  |
| **Total** | **133** | **100.0** |  |

| **Patient** | **MEF** | **NPS** | | **comment** |
| --- | --- | --- | --- | --- |
|  | **serotype** | **1^st^ Serotype** | **2^nd^ Serotype** |  |
| 4440 | b | b |  |  |
| 5325 | b | b |  |  |
| 5388 | b | b |  |  |
| 5936 | b | b |  |  |
| 6305 | b | b |  |  |
| 6310 | b | b |  |  |
| 7017 | b | b |  |  |
| 6947 | d | d |  |  |
| 6133 | e | e |  |  |
| 0624 | f | f |  |  |
| 4050 | f | f |  |  |
| 4449 | f | f |  |  |
| 5502 | f | f |  |  |
| 6952 | f | f |  |  |
| 8124 | f | f |  |  |
| 0112 | NT | NT |  |  |
| 0163 | NT | NT |  |  |
| 0201 | NT | NT |  |  |
| 0205 | NT | NT |  |  |
| 0307 | NT | NT |  |  |
| 0310 | NT | NT |  |  |
| 0637 | NT | NT |  |  |
| 0640 | NT | NT |  |  |
| 1141 | NT | NT |  |  |
| 1203 | NT | NT |  |  |
| 1403 | NT | NT |  |  |
| 1413 | NT | NT |  |  |
| 1524 | NT | NT |  |  |
| 1614 | NT | NT |  |  |
| 1619 | NT | NT |  |  |
| 1631 | NT | NT |  |  |
| 1707 | NT | NT |  |  |
| 1718 | NT | NT |  |  |
| 2012 | NT | NT |  |  |
| 2203 | NT | NT |  |  |
| 2206 | NT | NT |  |  |
| 2208 | NT | NT |  |  |
| 2349 | NT | NT |  |  |
| 2404 | NT | NT |  |  |
| 2513 | NT | NT |  |  |
| 2516 | NT | NT |  |  |
| 2532 | NT | NT |  |  |
| 3112 | NT | NT |  |  |
| 3211 | NT | NT |  |  |
| 3408 | NT | NT |  |  |
| 3427 | NT | NT |  |  |
| 3620 | NT | NT |  |  |
| 3643 | NT | NT |  |  |
| 3820 | NT | NT | f | 2 *H. influenzae* in NPS |
| 4002 | NT | NT |  |  |
| 4054 | NT | NT |  |  |
| 4072 | NT | NT |  |  |
| 4224 | NT | NT |  |  |
| 4240 | NT | NT |  |  |
| 4403 | NT | NT |  |  |
| 4410 | NT | NT |  |  |
| 4411 | NT | NT |  |  |
| 4431 | NT | NT |  |  |
| 4435 | NT | NT |  |  |
| 4441 | NT | NT |  |  |
| 4444 | NT | NT |  |  |
| 4720 | NT | NT |  |  |
| 4742 | NT | NT |  |  |
| 4802 | NT | NT |  |  |
| 4809 | NT | NT |  |  |
| 4813 | NT | NT |  |  |
| 5340 | NT | NT |  |  |
| 5402 | NT | NT |  |  |
| 5928 | NT | NT |  |  |
| 5937 | NT | NT |  |  |
| 6014 | NT | NT |  |  |
| 6301 | NT | NT |  |  |
| 6602 | NT | NT |  |  |
| 6721 | NT | NT |  |  |
| 7003 | NT | NT |  |  |
| 7302 | NT | NT |  |  |
| 7708 | NT | NT |  |  |
| 7817 | NT | NT |  |  |
| 8412 | NT | NT |  |  |
| 8501 | NT | NT |  |  |
| 1212 | b | f | NT | 2 *H. influenzae* in NPS |
| 0134 | b | NT |  |  |
| 2536 | b | NT |  |  |
| 3818 | b | NT |  |  |
| 3829 | b | NT |  |  |
| 4026 | b | NT |  |  |
| 8320 | b | NT |  |  |
| 0703 | d | NT |  |  |
| 5360 | d | NT |  |  |
| 5126 | e | f |  |  |
| 0145 | f | NT |  |  |
| 5125 | f | NT |  |  |
| 6008 | f | NT |  |  |
| 5114 | NT | b |  |  |
| 2864 | NT | f |  |  |
| 7001 | NT | f |  |  |
| 3224 |  | b |  | *H. influenzae* in MEF not serotyped |
| 5381 |  | f |  | *H. influenzae* in MEF not serotyped |
| 0103 |  |  |  | *H. influenzae* in MEF not serotyped |
| 0601 |  |  |  | *H. influenzae* in MEF not serotyped |
| 6727 | b |  |  | no *H. influenzae* in NPS |
| 1135 | f |  |  | no *H. influenzae* in NPS |
| 1701 | f |  |  | no *H. influenzae* in NPS |
| 4760 | f |  |  | no *H. influenzae* in NPS |
| 6917 | f |  |  | no *H. influenzae* in NPS |
| 8416 | f |  |  | no *H. influenzae* in NPS |
| 2344 | f |  |  | no NPS obtained |
| 2428 | f |  |  | no NPS obtained |
| 6991 | g |  |  | no *H. influenzae* in NPS |
| 0301 | NT |  |  | no *H. influenzae* in NPS |
| 0806 | NT |  |  | no *H. influenzae* in NPS |
| 1181 | NT |  |  | no *H. influenzae* in NPS |
| 2016 | NT |  |  | no *H. influenzae* in NPS |
| 2607 | NT |  |  | no *H. influenzae* in NPS |
| 2913 | NT |  |  | no *H. influenzae* in NPS |
| 3642 | NT |  |  | no *H. influenzae* in NPS |
| 3827 | NT |  |  | no *H. influenzae* in NPS |
| 4040 | NT |  |  | no *H. influenzae* in NPS |
| 4204 | NT |  |  | no *H. influenzae* in NPS |
| 4428 | NT |  |  | no *H. influenzae* in NPS |
| 4911 | NT |  |  | no *H. influenzae* in NPS |
| 5108 | NT |  |  | no *H. influenzae* in NPS |
| 5301 | NT |  |  | no *H. influenzae* in NPS |
| 5914 | NT |  |  | no *H. influenzae* in NPS |
| 6904 | NT |  |  | no *H. influenzae* in NPS |
| 7011 | NT |  |  | no *H. influenzae* in NPS |
| 7819 | NT |  |  | no *H. influenzae* in NPS |
| 0405 | NT |  |  | no NPS obtained |
| 1903 | NT |  |  | no NPS obtained |
| 2403 | NT |  |  | no NPS obtained |
| 3504 | NT |  |  | no NPS obtained |
| 3910 | NT |  |  | no NPS obtained |
| 3916 | NT |  |  | no NPS obtained |

| ***S. pyogenes* *emm* type** | **n=** | **%** | **% of NPS positive** |
| --- | --- | --- | --- |
| MEF=NPS | 197 | 62,5 | 99,0 |
| MEF≠NPS | 2 | 0,6 | 1,0 |
| no *emm* type in MEF | 9 | 2,9 |  |
| no *S. pyogenes* in NPS | 107 | 34,0 |  |
| **Total** | **315** | **100,0** |  |

| **Patient** | **MEF** | **NPS** | **comment** |
| --- | --- | --- | --- |
|  | ***emm* type** | ***emm* type** |  |
| 1501 | 1 | 1 |  |
| 3201 | 1 | 1 |  |
| 2101 | 1 | 1 |  |
| 3205 | 1 | 1 |  |
| 1802 | 1 | 1 |  |
| 4405 | 1 | 1 |  |
| 4212 | 1 | 1 |  |
| 2808 | 1 | 1 |  |
| 4732 | 1 | 1 |  |
| 2810 | 1 | 1 |  |
| 1410 | 1 | 1 |  |
| 1123 | 1 | 1 |  |
| 3417 | 1 | 1 |  |
| 4407 | 1 | 1 |  |
| 1209 | 1 | 1 |  |
| 3213 | 1 | 1 |  |
| 0620 | 1 | 1 |  |
| 0621 | 1 | 1 |  |
| 3631 | 1 | 1 |  |
| 3126 | 1 | 1 |  |
| 3637 | 1 | 1 |  |
| 2531 | 1 | 1 |  |
| 4421 | 1 | 1 |  |
| 7004 | 1 | 1 |  |
| 8303 | 1 | 1 |  |
| 5318 | 1 | 1 |  |
| 3644 | 1 | 1 |  |
| 5110 | 1 | 1 |  |
| 5914 | 1 | 1 |  |
| 5114 | 1 | 1 |  |
| 0832 | 1 | 1 |  |
| 4514 | 1 | 1 |  |
| 7705 | 1 | 1 |  |
| 0647 | 1 | 1 |  |
| 6944 | 1 | 1 |  |
| 8309 | 1 | 1 |  |
| 6411 | 1 | 1 |  |
| 8412 | 1 | 1 |  |
| 0659 | 1 | 1 |  |
| 8313 | 1 | 1 |  |
| 8414 | 1 | 1 |  |
| 5928 | 1 | 1 |  |
| 5338 | 1 | 1 |  |
| 7012 | 1 | 1 |  |
| 5343 | 1 | 1 |  |
| 4238 | 1 | 1 |  |
| 4439 | 1 | 1 |  |
| 4444 | 1 | 1 |  |
| 2394 | 1 | 1 |  |
| 7838 | 1 | 1 |  |
| 5373 | 1 | 1 |  |
| 8125 | 1 | 1 |  |
| 0162 | 1 | 1 |  |
| 6724 | 1 | 1 |  |
| 4452 | 1 | 1 |  |
| 7842 | 1 | 1 |  |
| 8127 | 1 | 1 |  |
| 5386 | 1 | 1 |  |
| 4455 | 1 | 1 |  |
| 5406 | 1 | 1 |  |
| 5128 | 1 | 1 |  |
| 4415 | 2 | 2 |  |
| 4418 | 2 | 2 |  |
| 6704 | 2 | 2 |  |
| 4423 | 2 | 2 |  |
| 4424 | 2 | 2 |  |
| 6130 | 2 | 2 |  |
| 4401 | 3 | 3 |  |
| 1713 | 3 | 3 |  |
| 3825 | 3 | 3 |  |
| 2623 | 3 | 3 |  |
| 0135 | 3 | 3 |  |
| 0632 | 3 | 3 |  |
| 5801 | 3 | 3 |  |
| 1224 | 3 | 3 |  |
| 6614 | 3 | 3 |  |
| 7109 | 3 | 3 |  |
| 3669 | 3 | 3 |  |
| 5209 | 3 | 3 |  |
| 7014 | 3 | 3 |  |
| 3225 | 3 | 3 |  |
| 4450 | 3 | 3 |  |
| 6139 | 3 | 3 |  |
| 0658 | 3 | 3 |  |
| 2840 | 4 | 4 |  |
| 0146 | 4 | 4 |  |
| 5331 | 4 | 4 |  |
| 2635 | 4 | 4 |  |
| 1169 | 5 | 5 |  |
| 3206 | 6 | 6 |  |
| 4302 | 6 | 6 |  |
| 2310 | 6 | 6 |  |
| 2614 | 6 | 6 |  |
| 3619 | 6 | 6 |  |
| 0628 | 6 | 6 |  |
| 0138 | 6 | 6 |  |
| 5315 | 6 | 6 |  |
| 5205 | 6 | 6 |  |
| 6408 | 6 | 6 |  |
| 3921 | 6 | 6 |  |
| 8312 | 6 | 6 |  |
| 4061 | 6 | 6 |  |
| 6809 | 6 | 6 |  |
| 6971 | 6 | 6 |  |
| 6717 | 6 | 6 |  |
| 6140 | 6 | 6 |  |
| 1401 | 12 | 12 |  |
| 3601 | 12 | 12 |  |
| 0107 | 12 | 12 |  |
| 2312 | 12 | 12 |  |
| 1710 | 12 | 12 |  |
| 3819 | 12 | 12 |  |
| 4222 | 12 | 12 |  |
| 1517 | 12 | 12 |  |
| 1818 | 12 | 12 |  |
| 5201 | 12 | 12 |  |
| 6302 | 12 | 12 |  |
| 7808 | 12 | 12 |  |
| 8002 | 12 | 12 |  |
| 6915 | 12 | 12 |  |
| 0639 | 12 | 12 |  |
| 4426 | 12 | 12 |  |
| 0829 | 12 | 12 |  |
| 4513 | 12 | 12 |  |
| 5915 | 12 | 12 |  |
| 7813 | 12 | 12 |  |
| 8110 | 12 | 12 |  |
| 8111 | 12 | 12 |  |
| 4524 | 12 | 12 |  |
| 3219 | 12 | 12 |  |
| 7822 | 12 | 12 |  |
| 4060 | 12 | 12 |  |
| 6954 | 12 | 12 |  |
| 6714 | 12 | 12 |  |
| 0154 | 12 | 12 |  |
| 2383 | 12 | 12 |  |
| 0667 | 12 | 12 |  |
| 2384 | 12 | 12 |  |
| 0157 | 12 | 12 |  |
| 5124 | 12 | 12 |  |
| 5357 | 12 | 12 |  |
| 4448 | 12 | 12 |  |
| 0684 | 12 | 12 |  |
| 1941 | 22 | 22 |  |
| 0602 | 28 | 28 |  |
| 0113 | 28 | 28 |  |
| 4913 | 28 | 28 |  |
| 8105 | 28 | 28 |  |
| 5011 | 28 | 28 |  |
| 1942 | 28 | 28 |  |
| 5907 | 28 | 28 |  |
| 5320 | 28 | 28 |  |
| 7103 | 28 | 28 |  |
| 6119 | 28 | 28 |  |
| 8113 | 28 | 28 |  |
| 5120 | 28 | 28 |  |
| 3222 | 28 | 28 |  |
| 3231 | 28 | 28 |  |
| 2878 | 28 | 28 |  |
| 2902 | 29 | 29 |  |
| 5115 | 29 | 29 |  |
| 4416 | 44 | 44 |  |
| 7707 | 44 | 44 |  |
| 4241 | 44 | 44 |  |
| 4453 | 48 | 48 |  |
| 1201 | 75 | 75 |  |
| 1504 | 75 | 75 |  |
| 4503 | 75 | 75 |  |
| 4807 | 75 | 75 |  |
| 4504 | 75 | 75 |  |
| 2622 | 75 | 75 |  |
| 1152 | 75 | 75 |  |
| 7812 | 75 | 75 |  |
| 7820 | 75 | 75 |  |
| 8306 | 75 | 75 |  |
| 2537 | 75 | 75 |  |
| 2386 | 75 | 75 |  |
| 4772 | 75 | 75 |  |
| 2605 | 77 | 77 |  |
| 2326 | 77 | 77 |  |
| 1302 | 89 | 89 |  |
| 4728 | 89 | 89 |  |
| 4213 | 89 | 89 |  |
| 1312 | 89 | 89 |  |
| 1207 | 89 | 89 |  |
| 6304 | 89 | 89 |  |
| 5113 | 89 | 89 |  |
| 5327 | 89 | 89 |  |
| 0651 | 89 | 89 |  |
| 0650 | 89 | 89 |  |
| 0654 | 89 | 89 |  |
| 0664 | 89 | 89 |  |
| 6310 | 89 | 89 |  |
| 1957 | 89 | 89 |  |
| 3223 | 89 | 89 |  |
| 3232 | 89 | 89 |  |
| 6141 | 89 | 89 |  |
| 2347 | 1 | 89 |  |
| 3009 | 3 | 6 |  |
| 2813 |  | 1 | *S. pyogenes* in MEF not *emm* typed |
| 1917 |  | 1 | *S. pyogenes* in MEF not *emm* typed |
| 5002 |  | 3 | *S. pyogenes* in MEF not *emm* typed |
| 6716 |  | 6 | *S. pyogenes* in MEF not *emm* typed |
| 6980 |  | 6 | *S. pyogenes* in MEF not *emm* typed |
| 2618 |  |  | *S. pyogenes* in MEF not *emm* typed, no *S. pyogenes* in NPS |
| 6127 |  |  | *S. pyogenes* in MEF not *emm* typed, no *S. pyogenes* in NPS |
| 2636 |  |  | *S. pyogenes* in MEF not *emm* typed, no *S. pyogenes* in NPS |
| 6722 |  |  | *S. pyogenes* in MEF not *emm* typed, no *S. pyogenes* in NPS |
| 4705 | 1 |  | no *S. pyogenes* in NPS |
| 3209 | 1 |  | no *S. pyogenes* in NPS |
| 1604 | 1 |  | no *S. pyogenes* in NPS |
| 0309 | 1 |  | no *S. pyogenes* in NPS |
| 0610 | 1 |  | no *S. pyogenes* in NPS |
| 2613 | 1 |  | no *S. pyogenes* in NPS |
| 3618 | 1 |  | no *S. pyogenes* in NPS |
| 3627 | 1 |  | no *S. pyogenes* in NPS |
| 3011 | 1 |  | no *S. pyogenes* in NPS |
| 0820 | 1 |  | no *S. pyogenes* in NPS |
| 3633 | 1 |  | no *S. pyogenes* in NPS |
| 1148 | 1 |  | no *S. pyogenes* in NPS |
| 7202 | 1 |  | no *S. pyogenes* in NPS |
| 8403 | 1 |  | no *S. pyogenes* in NPS |
| 1323 | 1 |  | no *S. pyogenes* in NPS |
| 4510 | 1 |  | no *S. pyogenes* in NPS |
| 6508 | 1 |  | no *S. pyogenes* in NPS |
| 0147 | 1 |  | no *S. pyogenes* in NPS |
| 2628 | 1 |  | no *S. pyogenes* in NPS |
| 1520 | 1 |  | no *S. pyogenes* in NPS |
| 1221 | 1 |  | no *S. pyogenes* in NPS |
| 6934 | 1 |  | no *S. pyogenes* in NPS |
| 1523 | 1 |  | no *S. pyogenes* in NPS |
| 6948 | 1 |  | no *S. pyogenes* in NPS |
| 6308 | 1 |  | no *S. pyogenes* in NPS |
| 3428 | 1 |  | no *S. pyogenes* in NPS |
| 3147 | 1 |  | no *S. pyogenes* in NPS |
| 6728 | 1 |  | no *S. pyogenes* in NPS |
| 0424 | 1 |  | no NPS obtained |
| 0437 | 1 |  | no NPS obtained |
| 1626 | 1 |  | no NPS obtained |
| 1628 | 1 |  | no NPS obtained |
| 2641 | 1 |  | no NPS obtained |
| 2434 | 1 |  | no NPS obtained |
| 2807 | 2 |  | no *S. pyogenes* in NPS |
| 1516 | 2 |  | no *S. pyogenes* in NPS |
| 1716 | 2 |  | no *S. pyogenes* in NPS |
| 3831 | 2 |  | no *S. pyogenes* in NPS |
| 1202 | 2 |  | no NPS obtained |
| 0144 | 3 |  | no *S. pyogenes* in NPS |
| 2852 | 3 |  | no *S. pyogenes* in NPS |
| 0661 | 3 |  | no *S. pyogenes* in NPS |
| 6977 | 3 |  | no *S. pyogenes* in NPS |
| 6016 | 3 |  | no NPS obtained |
| 3405 | 4 |  | no *S. pyogenes* in NPS |
| 3807 | 4 |  | no *S. pyogenes* in NPS |
| 4817 | 4 |  | no *S. pyogenes* in NPS |
| 2841 | 4 |  | no *S. pyogenes* in NPS |
| 1948 | 4 |  | no *S. pyogenes* in NPS |
| 5376 | 4 |  | no *S. pyogenes* in NPS |
| 4007 | 5 |  | no *S. pyogenes* in NPS |
| 0325 | 6 |  | no *S. pyogenes* in NPS |
| 2519 | 6 |  | no *S. pyogenes* in NPS |
| 0425 | 6 |  | no *S. pyogenes* in NPS |
| 4509 | 6 |  | no *S. pyogenes* in NPS |
| 1970 | 9 |  | no *S. pyogenes* in NPS |
| 0422 | 12 |  | no *S. pyogenes* in NPS |
| 6901 | 12 |  | no *S. pyogenes* in NPS |
| 0636 | 12 |  | no *S. pyogenes* in NPS |
| 5503 | 12 |  | no *S. pyogenes* in NPS |
| 5804 | 12 |  | no *S. pyogenes* in NPS |
| 4234 | 12 |  | no *S. pyogenes* in NPS |
| 6711 | 12 |  | no *S. pyogenes* in NPS |
| 3220 | 12 |  | no *S. pyogenes* in NPS |
| 1737 | 12 |  | no *S. pyogenes* in NPS |
| 8122 | 12 |  | no *S. pyogenes* in NPS |
| 2007 | 12 |  | no NPS obtained |
| 3508 | 12 |  | no NPS obtained |
| 6945 | 12 |  | no NPS obtained |
| 3514 | 12 |  | no NPS obtained |
| 0314 | 28 |  | no *S. pyogenes* in NPS |
| 0614 | 28 |  | no *S. pyogenes* in NPS |
| 4408 | 28 |  | no *S. pyogenes* in NPS |
| 0127 | 28 |  | no *S. pyogenes* in NPS |
| 0630 | 28 |  | no *S. pyogenes* in NPS |
| 1518 | 28 |  | no *S. pyogenes* in NPS |
| 8401 | 28 |  | no *S. pyogenes* in NPS |
| 5802 | 28 |  | no *S. pyogenes* in NPS |
| 6705 | 28 |  | no *S. pyogenes* in NPS |
| 5105 | 28 |  | no *S. pyogenes* in NPS |
| 1218 | 28 |  | no *S. pyogenes* in NPS |
| 6510 | 28 |  | no *S. pyogenes* in NPS |
| 8408 | 28 |  | no *S. pyogenes* in NPS |
| 0830 | 28 |  | no *S. pyogenes* in NPS |
| 2631 | 28 |  | no *S. pyogenes* in NPS |
| 6128 | 28 |  | no *S. pyogenes* in NPS |
| 3517 | 28 |  | no NPS obtained |
| 6972 | 29 |  | no *S. pyogenes* in NPS |
| 0803 | 75 |  | no *S. pyogenes* in NPS |
| 6902 | 75 |  | no *S. pyogenes* in NPS |
| 7809 | 75 |  | no *S. pyogenes* in NPS |
| 8406 | 75 |  | no *S. pyogenes* in NPS |
| 8108 | 75 |  | no *S. pyogenes* in NPS |
| 1973 | 75 |  | no *S. pyogenes* in NPS |
| 3003 | 77 |  | no *S. pyogenes* in NPS |
| 0116 | 87 |  | no *S. pyogenes* in NPS |
| 4711 | 89 |  | no *S. pyogenes* in NPS |
| 4730 | 89 |  | no *S. pyogenes* in NPS |
| 2616 | 89 |  | no *S. pyogenes* in NPS |
| 4816 | 89 |  | no *S. pyogenes* in NPS |
| 4039 | 89 |  | no *S. pyogenes* in NPS |
| 3124 | 89 |  | no *S. pyogenes* in NPS |
| 3131 | 89 |  | no *S. pyogenes* in NPS |
| 4058 | 89 |  | no *S. pyogenes* in NPS |
| 3664 | 89 |  | no *S. pyogenes* in NPS |
| 2003 | 89 |  | no NPS obtained |
| 2881 | 118 |  | no *S. pyogenes* in NPS |

**Supplemental material 4:** Vaccination status of patients included in the study.

| **vaccination status** | **vaccine** | | **year1** | **year2** | **year3** | **year4** | **year5** | **year6** | **year7** | **total** |
| --- | --- | --- | --- | --- | --- | --- | --- | --- | --- | --- |
| vaccinated | PCV7 | | 323 | 213 | 103 | 93 | 25 |  |  | 757 |
| vaccinated | PCV10 | | 1 | 13 | 8 | 24 | 19 | 11 | 7 | 83 |
| vaccinated | PCV13 | |  | 11 | 47 | 183 | 209 | 207 | 185 | 842 |
| vaccinated | PCV7/PCV10 | |  | 8 | 2 | 13 | 4 |  |  | 27 |
| vaccinated | PCV7/PCV13 | |  | 9 | 18 | 57 | 39 | 9 |  | 132 |
| vaccinated | PCV10/PCV13 | |  | 2 | 2 | 7 | 5 | 5 |  | 21 |
| vaccinated | PCV7/PCV10/PCV13 | |  |  |  | 1 |  |  |  | 1 |
| vaccinated | PCV13/PCV10/PCV13 | |  |  |  |  |  | 1 | 1 | 2 |
| vaccinated | vaccine unknown | | 3 | 6 | 2 | 1 | 1 | 2 | 4 | 19 |
| **vaccinated total** |  | | **327** | **262** | **182** | **379** | **302** | **235** | **197** | **1884** |
| not vaccinated | no | | 111 | 35 | 27 | 22 | 27 | 10 | 12 | 244 |
| unknown | unknown | | 1 | 3 |  | 3 | 3 |  |  | 10 |
|  | **total** | | **439** | **300** | **209** | **404** | **332** | **245** | **209** | **2138** |
|  |  | |  |  |  |  |  |  |  |  |
| % vaccinated |  | | 74,5 | 87,3 | 87,1 | 93,8 | 91,0 | 95,9 | 94,3 | 88,1 |
| % PCV7 |  | | 98,8 | 81,3 | 56,6 | 24,5 | 8,3 | 0,0 | 0,0 | 40,2 |
| % PCV10 |  | | 0,3 | 5,0 | 4,4 | 6,3 | 6,3 | 4,7 | 3,6 | 4,4 |
| % PCV13 |  | | 0,0 | 4,2 | 25,8 | 48,3 | 69,2 | 88,1 | 93,9 | 44,7 |
| % PCV7/PCV10 |  | | 0,0 | 3,1 | 1,1 | 3,4 | 1,3 | 0,0 | 0,0 | 1,4 |
| % PCV7/PCV13 |  | | 0,0 | 3,4 | 9,9 | 15,0 | 12,9 | 3,8 | 0,0 | 7,0 |
| % PCV7/PCV10/PCV13 |  | | 0,0 | 0,0 | 0,0 | 0,3 | 0,0 | 0,0 | 0,0 | 0,1 |
| % PCV10/PCV13 |  | | 0,0 | 0,8 | 1,1 | 1,8 | 1,7 | 2,1 | 0,0 | 1,1 |
| % PCV13/PCV10/PCV13 |  | | 0,0 | 0,0 | 0,0 | 0,0 | 0,0 | 0,4 | 0,5 | 0,1 |
| % vaccine unknown |  | | 0,9 | 2,3 | 1,1 | 0,3 | 0,3 | 0,9 | 2,0 | 1,0 |
|  | **total** | | **100,0** | **100,0** | **100,0** | **100,0** | **100,0** | **100,0** | **100,0** | **100,0** |
|  |  | |  |  |  |  |  |  |  |  |
| **vaccination status** | **n=** | | **%** |  |  |  |  |  |  |  |
| vaccinated | 1884 | | 88,1 |  |  |  |  |  |  |  |
| not vaccinated | 244 | | 11,4 |  |  |  |  |  |  |  |
| vaccination status unknown | 10 | | 0,5 |  |  |  |  |  |  |  |
| **total** | **2138** | | **100,0** |  |  |  |  |  |  |  |
|  | |  |  |  |  |  |  |  |  |  |
| **vaccination according to age** | | **n=** | **%** |  |  |  |  |  |  |  |
| yes | | 1544 | 82,0 |  |  |  |  |  |  |  |
| no | | 326 | 17,3 |  |  |  |  |  |  |  |
| unknown | | 14 | 0,7 |  |  |  |  |  |  |  |
| **Total** | | **1884** | **100,0** |  |  |  |  |  |  |  |
|  |  | |  |  |  |  |  |  |  |  |
| **vaccine** | **n=** | | **%** |  |  |  |  |  |  |  |
| PCV7 | 757 | | 40,2 |  |  |  |  |  |  |  |
| PCV10 | 83 | | 4,4 |  |  |  |  |  |  |  |
| PCV13 | 842 | | 44,7 |  |  |  |  |  |  |  |
| PCV7/PCV10 | 27 | | 1,4 |  |  |  |  |  |  |  |
| PCV7/PCV13 | 132 | | 7,0 |  |  |  |  |  |  |  |
| PCV10/PCV13 | 21 | | 1,1 |  |  |  |  |  |  |  |
| PCV7/PCV10/PCV13 | 1 | | 0,1 |  |  |  |  |  |  |  |
| PCV13/PCV10/PCV13 | 2 | | 0,1 |  |  |  |  |  |  |  |
| vaccine unknown | 19 | | 1,0 |  |  |  |  |  |  |  |
| **total** | **1884** | | **100,0** |  |  |  |  |  |  |  |

**Supplemental material 5:** Interspecies and demographic associations for patients with severe spontaneously ruptured AOM in Germany. Extrema in yellow.

| Yearly counts of children with acute otitis media living with self-admitted in-home tobacco smokers | | | | | | | | | | | | | | | | |
| --- | --- | --- | --- | --- | --- | --- | --- | --- | --- | --- | --- | --- | --- | --- | --- | --- |
| All Pathogens | | | | | | | | | | | | | | | | |
| Number of smokers | Y1 | | Y2 | | Y3 | | Y4 | | Y5 | | Y6 | | Y7 | | Total | |
|  | n | % | n | % | n | % | n | % | n | % | n | % | n | % | n | % |
| 0 | 337 | 79% | 246 | 82% | 170 | 82% | 314 | 78% | 278 | 83% | 206 | 84% | 173 | 83% | 1732 | 81% |
| 1 | 60 | 14% | 36 | 12% | 19 | 9% | 58 | 14% | 38 | 11% | 24 | 10% | 25 | 12% | 262 | 12% |
| 2 | 28 | 7% | 17 | 6% | 18 | 9% | 28 | 7% | 17 | 5% | 14 | 6% | 11 | 5% | 134 | 6% |
| 3 | 2 | 0% |  | 0% |  | 0% | 2 | 0% |  | 0% |  | 0% |  | 0% | 4 | 0% |
| Yearly Total | 427 | 100% | 299 | 100% | 207 | 100% | 402 | 100% | 333 | 100% | 244 | 100% | 209 | 100% | 2132 | 100% |
| AtLeast1 | 90 | 21% | 53 | 18% | 37 | 18% | 88 | 22% | 55 | 17% | 38 | 16% | 36 | 17% | 400 | 19% |
| *Streptococcus pneumoniae* | | | | | | | | | | | | | | | | |
| Number of smokers | Y1 |  | Y2 |  | Y3 |  | Y4 |  | Y5 |  | Y6 |  | Y7 |  | Total |  |
|  | n | % | n | % | n | % | n | % | n | % | n | % | n | % | n | % |
| 0 | 28 | 74% | 21 | 64% | 9 | 75% | 20 | 74% | 19 | 90% | 15 | 94% | 8 | 67% | 121 | 75% |
| 1 | 8 | 21% | 9 | 27% | 2 | 17% | 5 | 19% | 2 | 10% | 1 | 6% | 1 | 8% | 29 | 18% |
| 2 | 2 | 5% | 3 | 9% | 1 | 8% | 1 | 4% |  | 0% |  | 0% | 3 | 25% | 10 | 6% |
| 3 |  | 0% |  | 0% |  | 0% | 1 | 4% |  | 0% |  | 0% |  | 0% | 1 | 1% |
| Yearly Total | 38 | 100% | 33 | 100% | 12 | 100% | 27 | 100% | 21 | 100% | 16 | 100% | 12 | 100% | 161 | 100% |
| AtLeast1 | 10 | 26% | 12 | 36% | 3 | 25% | 7 | 26% | 2 | 10% | 1 | 6% | 4 | 33% | 40 | 25% |
| *Streptococcus pyogenes* | | | | | | | | | | | | | | | | |
| Number of smokers | Y1 |  | Y2 |  | Y3 |  | Y4 |  | Y5 |  | Y6 |  | Y7 |  | Total |  |
|  | n | % | n | % | n | % | n | % | n | % | n | % | n | % | n | % |
| 0 | 43 | 81% | 29 | 85% | 21 | 91% | 54 | 74% | 50 | 86% | 33 | 97% | 20 | 63% | 250 | 81% |
| 1 | 6 | 11% | 4 | 12% | 1 | 4% | 11 | 15% | 6 | 10% | 1 | 3% | 8 | 25% | 37 | 12% |
| 2 | 4 | 8% | 1 | 3% | 1 | 4% | 8 | 11% | 2 | 3% |  | 0% | 4 | 13% | 20 | 7% |
| Yearly Total | 53 | 100% | 34 | 100% | 23 | 100% | 73 | 100% | 58 | 100% | 34 | 100% | 32 | 100% | 307 | 100% |
| AtLeast1 | 10 | 19% | 5 | 15% | 2 | 9% | 19 | 26% | 8 | 14% | 1 | 3% | 12 | 38% | 57 | 19% |
| *Haemophilus influenzae* | | | | | | | | | | | | | | | | |
| Number of smokers | Y1 |  | Y2 |  | Y3 |  | Y4 |  | Y5 |  | Y6 |  | Y7 |  | Total |  |
|  | n | % | n | % | n | % | n | % | n | % | n | % | n | % | n | % |
| 0 | 23 | 79% | 13 | 87% | 12 | 86% | 21 | 88% | 12 | 86% | 11 | 73% | 9 | 75% | 101 | 82% |
| 1 | 5 | 17% | 1 | 7% | 1 | 7% | 2 | 8% | 2 | 14% | 3 | 20% | 3 | 25% | 17 | 14% |
| 2 |  | 0% | 1 | 7% | 1 | 7% | 1 | 4% |  | 0% | 1 | 7% |  | 0% | 4 | 3% |
| 3 | 1 | 3% |  | 0% |  | 0% |  | 0% |  | 0% |  | 0% |  | 0% | 1 | 1% |
| Yearly Total | 29 | 100% | 15 | 100% | 14 | 100% | 24 | 100% | 14 | 100% | 15 | 100% | 12 | 100% | 123 | 100% |
| AtLeast1 | 6 | 21% | 2 | 13% | 2 | 14% | 3 | 13% | 2 | 14% | 4 | 27% | 3 | 25% | 22 | 18% |
| *Moraxella catarrhalis* | | | | | | | | | | | | | | | | |
| Number of smokers | Y1 |  | Y2 |  | Y3 |  | Y4 |  | Y5 |  | Y6 |  | Y7 |  | Total |  |
|  | n | % | n | % | n | % | n | % | n | % | n | % | n | % | n | % |
| 0 | 5 | 83% |  |  |  |  | 1 | 100% |  |  |  |  |  |  | 6 | 86% |
| 1 | 1 | 17% |  |  |  |  |  | 0% |  |  |  |  |  |  | 1 | 14% |
| Yearly Total | 6 | 100% |  |  |  |  | 1 | 100% |  |  |  |  |  |  | 7 | 100% |

| Yearly counts of children with acute otitis media who attended community daycare | | | | | | | | | | | | | | | | |
| --- | --- | --- | --- | --- | --- | --- | --- | --- | --- | --- | --- | --- | --- | --- | --- | --- |
| All Pathogens | | | | | | | | | | | | | | | | |
| Attended daycare? | Y1 | | Y2 | | Y3 | | Y4 | | Y5 | | Y6 | | Y7 | | Total | |
|  | n | % | n | % | n | % | n | % | n | % | n | % | n | % | n | % |
| No | 193 | 45% | 133 | 45% | 82 | 40% | 149 | 37% | 126 | 38% | 90 | 37% | 90 | 43% | 863 | 41% |
| Yes | 232 | 55% | 165 | 55% | 125 | 60% | 255 | 63% | 207 | 62% | 155 | 63% | 119 | 57% | 1258 | 59% |
| Yearly Total | 425 | 100% | 298 | 100% | 207 | 100% | 404 | 100% | 333 | 100% | 245 | 100% | 209 | 100% | 2121 | 100% |
| *Streptococcus pneumoniae* | | | | | | | | | | | | | | | | |
| Attended daycare? | Y1 | | Y2 | | Y3 | | Y4 | | Y5 | | Y6 | | Y7 | | Total | |
|  | n | % | n | % | n | % | n | % | n | % | n | % | n | % | n | % |
| No | 16 | 42% | 12 | 36% | 4 | 33% | 12 | 44% | 9 | 43% | 6 | 38% | 4 | 33% | 63 | 40% |
| Yes | 22 | 58% | 21 | 64% | 8 | 67% | 15 | 56% | 12 | 57% | 10 | 63% | 8 | 67% | 96 | 60% |
| Yearly Total | 38 | 100% | 33 | 100% | 12 | 100% | 27 | 100% | 21 | 100% | 16 | 100% | 12 | 100% | 159 | 100% |
| *Streptococcus pyogenes* | | | | | | | | | | | | | | | | |
| Attended daycare? | Y1 | | Y2 | | Y3 | | Y4 | | Y5 | | Y6 | | Y7 | | Total | |
|  | n | % | n | % | n | % | n | % | n | % | n | % | n | % | n | % |
| No | 25 | 47% | 17 | 50% | 8 | 35% | 25 | 34% | 20 | 34% | 14 | 40% | 11 | 34% | 120 | 39% |
| Yes | 28 | 53% | 17 | 50% | 15 | 65% | 48 | 66% | 38 | 66% | 21 | 60% | 21 | 66% | 188 | 61% |
| Yearly Total | 53 | 100% | 34 | 100% | 23 | 100% | 73 | 100% | 58 | 100% | 35 | 100% | 32 | 100% | 308 | 100% |
| *Haemophilus influenzae* | | | | | | | | | | | | | | | | |
| Attended daycare? | Y1 | | Y2 | | Y3 | | Y4 | | Y5 | | Y6 | | Y7 | | Total | |
|  | n | % | n | % | n | % | n | % | n | % | n | % | n | % | n | % |
| No | 16 | 55% | 4 | 27% | 5 | 36% | 8 | 33% | 5 | 36% | 5 | 33% | 3 | 25% | 46 | 37% |
| Yes | 13 | 45% | 11 | 73% | 9 | 64% | 16 | 67% | 9 | 64% | 10 | 67% | 9 | 75% | 77 | 63% |
| Yearly Total | 29 | 100% | 15 | 100% | 14 | 100% | 24 | 100% | 14 | 100% | 15 | 100% | 12 | 100% | 123 | 100% |
| *Moraxella catarrhalis* | | | | | | | | | | | | | | | | |
| Attended daycare? | Y1 | | Y2 | | Y3 | | Y4 | | Y5 | | Y6 | | Y7 | | Total | |
|  | n | % | n | % | n | % | n | % | n | % | n | % | n | % | n | % |
| No | 1 | 17% |  |  |  |  | 1 | 100% |  |  |  |  |  |  | 2 | 29% |
| Yes | 5 | 83% |  |  |  |  |  | 0% |  |  |  |  |  |  | 5 | 71% |
| Yearly Total | 6 | 100% |  |  |  |  | 1 | 100% |  |  |  |  |  |  | 7 | 100% |

| Yearly counts of children with acute otitis media who had siblings in the household | | | | | | | | | | | | | | | | |
| --- | --- | --- | --- | --- | --- | --- | --- | --- | --- | --- | --- | --- | --- | --- | --- | --- |
| All Pathogens | | | | | | | | | | | | | | | | |
| Number of Siblings | Y1 | | Y2 | | Y3 | | Y4 | | Y5 | | Y6 | | Y7 | | Total | |
|  | n | % | n | % | n | % | n | % | n | % | n | % | n | % | n | % |
| 0 | 128 | 30% | 87 | 29% | 74 | 36% | 121 | 30% | 92 | 28% | 73 | 30% | 74 | 35% | 649 | 31% |
| 1 | 193 | 45% | 147 | 49% | 88 | 43% | 189 | 47% | 160 | 48% | 129 | 53% | 85 | 41% | 991 | 47% |
| 2 | 68 | 16% | 45 | 15% | 30 | 15% | 65 | 16% | 59 | 18% | 26 | 11% | 30 | 14% | 323 | 15% |
| 3 | 29 | 7% | 16 | 5% | 8 | 4% | 17 | 4% | 16 | 5% | 11 | 4% | 11 | 5% | 108 | 5% |
| 4 | 3 | 1% | 1 | 0% | 5 | 2% | 7 | 2% | 2 | 1% | 5 | 2% | 6 | 3% | 29 | 1% |
| 5 | 3 | 1% | 2 | 1% | 1 | 0% | 2 | 0% | 4 | 1% | 1 | 0% | 2 | 1% | 15 | 1% |
| 6 | 1 | 0% |  | 0% |  | 0% | 1 | 0% |  | 0% |  | 0% | 1 | 0% | 3 | 0% |
| 7 | 1 | 0% | 1 | 0% |  | 0% | 1 | 0% |  | 0% |  | 0% |  | 0% | 3 | 0% |
| 8 |  | 0% |  | 0% |  | 0% | 1 | 0% |  | 0% |  | 0% |  | 0% | 1 | 0% |
| 9 |  | 0% | 1 | 0% |  | 0% |  | 0% |  | 0% |  | 0% |  | 0% | 1 | 0% |
| Yearly Total | 426 | 100% | 300 | 100% | 206 | 100% | 404 | 100% | 333 | 100% | 245 | 100% | 209 | 100% | 2123 | 100% |
| AtLeast1 | 298 | 70% | 213 | 71% | 132 | 64% | 283 | 70% | 241 | 72% | 172 | 70% | 135 | 65% | 1474 | 69% |
| *Streptococcus pneumoniae* | | | | | | | | | | | | | | | | |
| Number of Siblings | Y1 | | Y2 | | Y3 | | Y4 | | Y5 | | Y6 | | Y7 | | Total | |
|  | n | % | n | % | n | % | n | % | n | % | n | % | n | % | n | % |
| 0 | 18 | 47% | 10 | 30% | 5 | 42% | 9 | 33% | 9 | 43% | 6 | 38% | 4 | 33% | 61 | 38% |
| 1 | 14 | 37% | 18 | 55% | 6 | 50% | 14 | 52% | 10 | 48% | 7 | 44% | 7 | 58% | 76 | 48% |
| 2 | 3 | 8% | 3 | 9% |  | 0% | 3 | 11% | 1 | 5% | 1 | 6% |  | 0% | 11 | 7% |
| 3 | 3 | 8% | 2 | 6% |  | 0% |  | 0% | 1 | 5% |  | 0% | 1 | 8% | 7 | 4% |
| 4 |  | 0% |  | 0% | 1 | 8% |  | 0% |  | 0% | 1 | 6% |  | 0% | 2 | 1% |
| 5 |  | 0% |  | 0% |  | 0% | 1 | 4% |  | 0% | 1 | 6% |  | 0% | 2 | 1% |
| Yearly Total | 38 | 100% | 33 | 100% | 12 | 100% | 27 | 100% | 21 | 100% | 16 | 100% | 12 | 100% | 159 | 100% |
| AtLeast1 | 20 | 53% | 23 | 70% | 7 | 58% | 18 | 67% | 12 | 57% | 10 | 63% | 8 | 67% | 98 | 62% |
| *Streptococcus pyogenes* | | | | | | | | | | | | | | | | |
| Number of Siblings | Y1 | | Y2 | | Y3 | | Y4 | | Y5 | | Y6 | | Y7 | | Total | |
|  | n | % | n | % | n | % | n | % | n | % | n | % | n | % | n | % |
| 0 | 12 | 23% | 10 | 29% | 4 | 17% | 23 | 32% | 13 | 22% | 13 | 37% | 10 | 31% | 85 | 28% |
| 1 | 24 | 45% | 13 | 38% | 11 | 48% | 29 | 40% | 31 | 53% | 13 | 37% | 15 | 47% | 136 | 44% |
| 2 | 12 | 23% | 8 | 24% | 7 | 30% | 14 | 19% | 11 | 19% | 6 | 17% | 5 | 16% | 63 | 20% |
| 3 | 2 | 4% | 2 | 6% |  | 0% | 4 | 5% | 1 | 2% | 2 | 6% | 1 | 3% | 12 | 4% |
| 4 | 1 | 2% | 1 | 3% | 1 | 4% | 2 | 3% |  | 0% | 1 | 3% |  | 0% | 6 | 2% |
| 5 | 1 | 2% |  | 0% |  | 0% | 1 | 1% | 2 | 3% |  | 0% | 1 | 3% | 5 | 2% |
| 6 | 1 | 2% |  | 0% |  | 0% |  | 0% |  | 0% |  | 0% |  | 0% | 1 | 0% |
| Yearly Total | 53 | 100% | 34 | 100% | 23 | 100% | 73 | 100% | 58 | 100% | 35 | 100% | 32 | 100% | 308 | 100% |
| AtLeast1 | 41 | 77% | 24 | 71% | 19 | 83% | 50 | 68% | 45 | 78% | 22 | 63% | 22 | 69% | 223 | 72% |
| *Haemophilus influenzae* | | | | | | | | | | | | | | | | |
| Number of Siblings | Y1 | | Y2 | | Y3 | | Y4 | | Y5 | | Y6 | | Y7 | | Total | |
|  | n | % | n | % | n | % | n | % | n | % | n | % | n | % | n | % |
| 0 | 6 | 21% | 5 | 33% | 7 | 50% | 6 | 25% | 3 | 21% | 3 | 20% | 2 | 17% | 32 | 26% |
| 1 | 18 | 62% | 7 | 47% | 5 | 36% | 12 | 50% | 7 | 50% | 7 | 47% | 6 | 50% | 62 | 50% |
| 2 | 2 | 7% | 3 | 20% | 1 | 7% | 2 | 8% | 3 | 21% | 3 | 20% | 1 | 8% | 15 | 12% |
| 3 | 1 | 3% |  | 0% | 1 | 7% | 2 | 8% | 1 | 7% | 1 | 7% | 1 | 8% | 7 | 6% |
| 4 | 1 | 3% |  | 0% |  | 0% | 1 | 4% |  | 0% | 1 | 7% | 2 | 17% | 5 | 4% |
| 5 | 1 | 3% |  | 0% |  | 0% |  | 0% |  | 0% |  | 0% |  | 0% | 1 | 1% |
| 6 |  | 0% |  | 0% |  | 0% | 1 | 4% |  | 0% |  | 0% |  | 0% | 1 | 1% |
| Yearly Total | 29 | 100% | 15 | 100% | 14 | 100% | 24 | 100% | 14 | 100% | 15 | 100% | 12 | 100% | 123 | 100% |
| AtLeast1 | 23 | 79% | 10 | 67% | 7 | 50% | 18 | 75% | 11 | 79% | 12 | 80% | 10 | 83% | 91 | 74% |
| *Moraxella catarrhalis* | | | | | | | | | | | | | | | | |
| Number of Siblings | Y1 | | Y2 | | Y3 | | Y4 | | Y5 | | Y6 | | Y7 | | Total | |
|  | n | % | n | % | n | % | n | % | n | % | n | % | n | % | n | % |
| 0 | 2 | 33% |  |  |  |  | 1 | 100% |  |  |  |  |  |  | 3 | 43% |
| 1 | 3 | 50% |  |  |  |  |  | 0% |  |  |  |  |  |  | 3 | 43% |
| 2 | 1 | 17% |  |  |  |  |  | 0% |  |  |  |  |  |  | 1 | 14% |
| Yearly Total | 6 | 100% |  |  |  |  | 1 | 100% |  |  |  |  |  |  | 7 | 100% |
| AtLeast1 | 4 | 67% |  |  |  |  | 0 | 0% |  |  |  |  |  |  | 4 | 57% |

| Yearly count of children with acute otitis media who had siblings in community daycare | | | | | | | | | | | | | | | | |
| --- | --- | --- | --- | --- | --- | --- | --- | --- | --- | --- | --- | --- | --- | --- | --- | --- |
| All Pathogens | | | | | | | | | | | | | | | | |
| Number of Siblings in Daycare | Y1 | | Y2 | | Y3 | | Y4 | | Y5 | | Y6 | | Y7 | | Total | |
|  | n | % | n | % | n | % | n | % | n | % | n | % | n | % | n | % |
| 0 | 176 | 42% | 115 | 39% | 100 | 49% | 166 | 41% | 138 | 42% | 100 | 41% | 93 | 44% | 888 | 42% |
| 1 | 175 | 41% | 127 | 43% | 69 | 34% | 158 | 39% | 125 | 38% | 104 | 42% | 74 | 35% | 832 | 39% |
| 2 | 49 | 12% | 40 | 14% | 22 | 11% | 63 | 16% | 50 | 15% | 26 | 11% | 27 | 13% | 277 | 13% |
| 3 | 22 | 5% | 12 | 4% | 11 | 5% | 15 | 4% | 18 | 5% | 14 | 6% | 15 | 7% | 107 | 5% |
| 4 | 1 | 0% |  | 0% | 1 | 0% |  | 0% |  | 0% | 1 | 0% |  | 0% | 3 | 0% |
| Yearly Total | 423 | 100% | 294 | 100% | 203 | 100% | 402 | 100% | 331 | 100% | 245 | 100% | 209 | 100% | 2109 | 100% |
| AtLeast1 | 247 | 58% | 179 | 61% | 103 | 51% | 236 | 59% | 193 | 58% | 145 | 59% | 116 | 56% | 1219 | 58% |
| *Streptococcus pneumoniae* | | | | | | | | | | | | | | | | |
| Number of Siblings in Daycare | Y1 | | Y2 | | Y3 | | Y4 | | Y5 | | Y6 | | Y7 | | Total | |
|  | n | % | n | % | n | % | n | % | n | % | n | % | n | % | n | % |
| 0 | 24 | 63% | 12 | 39% | 7 | 64% | 13 | 48% | 11 | 52% | 10 | 63% | 5 | 42% | 82 | 53% |
| 1 | 9 | 24% | 15 | 48% | 3 | 27% | 10 | 37% | 8 | 38% | 3 | 19% | 6 | 50% | 54 | 35% |
| 2 | 4 | 11% | 4 | 13% |  | 0% | 3 | 11% | 2 | 10% | 1 | 6% |  | 0% | 14 | 9% |
| 3 | 1 | 3% |  | 0% | 1 | 9% | 1 | 4% |  | 0% | 2 | 13% | 1 | 8% | 6 | 4% |
| Yearly Total | 38 | 100% | 31 | 100% | 11 | 100% | 27 | 100% | 21 | 100% | 16 | 100% | 12 | 100% | 156 | 100% |
| AtLeast1 | 14 | 37% | 19 | 61% | 4 | 36% | 14 | 52% | 10 | 48% | 6 | 38% | 7 | 58% | 74 | 47% |
| *Streptococcus pyogenes* | | | | | | | | | | | | | | | | |
| Number of Siblings in Daycare | Y1 | | Y2 | | Y3 | | Y4 | | Y5 | | Y6 | | Y7 | | Total | |
|  | n | % | n | % | n | % | n | % | n | % | n | % | n | % | n | % |
| 0 | 17 | 32% | 15 | 44% | 5 | 23% | 34 | 47% | 21 | 36% | 15 | 43% | 11 | 34% | 118 | 39% |
| 1 | 23 | 43% | 10 | 29% | 12 | 55% | 20 | 28% | 25 | 43% | 11 | 31% | 16 | 50% | 117 | 38% |
| 2 | 10 | 19% | 7 | 21% | 4 | 18% | 14 | 19% | 9 | 16% | 6 | 17% | 3 | 9% | 53 | 17% |
| 3 | 2 | 4% | 2 | 6% | 1 | 5% | 4 | 6% | 3 | 5% | 3 | 9% | 2 | 6% | 17 | 6% |
| 4 | 1 | 2% |  | 0% |  | 0% |  | 0% |  | 0% |  | 0% |  | 0% | 1 | 0% |
| Yearly Total | 53 | 100% | 34 | 100% | 22 | 100% | 72 | 100% | 58 | 100% | 35 | 100% | 32 | 100% | 306 | 100% |
| AtLeast1 | 36 | 68% | 19 | 56% | 17 | 77% | 38 | 53% | 37 | 64% | 20 | 57% | 21 | 66% | 188 | 61% |
| *Haemophilus influenzae* | | | | | | | | | | | | | | | | |
| Number of Siblings in Daycare | Y1 | | Y2 | | Y3 | | Y4 | | Y5 | | Y6 | | Y7 | | Total | |
|  | n | % | n | % | n | % | n | % | n | % | n | % | n | % | n | % |
| 0 | 9 | 31% | 7 | 47% | 9 | 64% | 7 | 30% | 3 | 21% | 5 | 33% | 3 | 25% | 43 | 35% |
| 1 | 17 | 59% | 7 | 47% | 4 | 29% | 10 | 43% | 8 | 57% | 5 | 33% | 5 | 42% | 56 | 46% |
| 2 | 1 | 3% | 1 | 7% |  | 0% | 3 | 13% | 2 | 14% | 3 | 20% | 1 | 8% | 11 | 9% |
| 3 | 2 | 7% |  | 0% | 1 | 7% | 3 | 13% | 1 | 7% | 2 | 13% | 3 | 25% | 12 | 10% |
| Yearly Total | 29 | 100% | 15 | 100% | 14 | 100% | 23 | 100% | 14 | 100% | 15 | 100% | 12 | 100% | 122 | 100% |
| AtLeast1 | 20 | 69% | 8 | 53% | 5 | 36% | 16 | 70% | 11 | 79% | 10 | 67% | 9 | 75% | 79 | 65% |
| *Moraxella catarrhalis* | | | | | | | | | | | | | | | | |
| Number of Siblings in Daycare | Y1 | | Y2 | | Y3 | | Y4 | | Y5 | | Y6 | | Y7 | | Total | |
|  | n | % | n | % | n | % | n | % | n | % | n | % | n | % | n | % |
| 0 | 4 | 67% |  |  |  |  | 1 | 100% |  |  |  |  |  |  | 5 | 71% |
| 1 | 2 | 33% |  |  |  |  |  | 0% |  |  |  |  |  |  | 2 | 29% |
| Yearly Total | 6 | 100% |  |  |  |  | 1 | 100% |  |  |  |  |  |  | 7 | 100% |
